# Efficacy and safety of GSK3772847 in participants with moderate-to-severe asthma with allergic fungal airway disease: A phase IIa randomized, multicenter, double-blind, sponsor-open, comparative trial

**DOI:** 10.1371/journal.pone.0281205

**Published:** 2023-02-03

**Authors:** Chika Akinseye, Courtney Crim, Amy Newlands, David Fairman

**Affiliations:** 1 Medicines Research Centre, GSK, Stevenage, Hertfordshire, United Kingdom; 2 Clinical Sciences–Respiratory, GSK, Research Triangle Park, North Carolina, United States of America; 3 Respiratory Medicines Development Centre, GSK, Brentford, Middlesex, United Kingdom; 4 Clinical Pharmacology Modelling and Simulation, GSK, Stevenage, Hertfordshire, United Kingdom; National Cancer Centre Singapore, SINGAPORE

## Abstract

**Introduction:**

Current treatments for allergic fungal airway disease are not specific for asthma and are associated with limited efficacy or safety concerns. This Phase IIa randomized, multicenter, double-blind, sponsor-open, comparative trial assessed the efficacy and safety of GSK3772847, an anti-interleukin-33 receptor monoclonal antibody, in moderate-to-severe asthma patients with allergic fungal airway disease (ClinicalTrials.gov: NCT03393806).

**Methods:**

Key inclusion criteria required participants of ≥18 years of age with a documented diagnosis of moderate-to-severe asthma (≥12 months) treated with inhaled corticosteroid and long-acting β2-agonist (≥4 months); evidence of allergic fungal airway disease (fungal sensitization to *Aspergillus fumigatus* [>0.35 KU/L] or *Penicillium chrysogenum* [>0.35 KU/L] and no history of concurrent respiratory disease/recurrent or ongoing non-pulmonary infections. Participants were randomized (1:1) to GSK3772847 (10 mg/kg) or matching placebo intravenously administered at Weeks 0, 4, and 8, in addition to standard of care. Randomization was based on systemic anti-fungal treatment status at screening. Primary endpoints were change from baseline (Week 0) to Week 12 in blood eosinophils and fractional exhaled nitric oxide.

**Results:**

Participants (n = 17) were randomized to GSK3772847 (n = 8) or placebo (n = 9) for 12 weeks and included in efficacy and safety analyses. This study was terminated early due to the high rate of screen failure, low enrollment, and unlikely feasibility of timely study completion. There were no differences observed in blood eosinophils or fractional exhaled nitric oxide between treatment arms. Target engagement was demonstrated by reductions in free soluble suppressor of tumorigenicity 2 levels in the GSK3772847 arm throughout the treatment period. No deaths occurred and no new safety signals were identified.

**Conclusions:**

Lack of clinical benefits with GSK3772847 was likely due to the small sample size, highlighting the need for larger prospective studies.

## Introduction

Thermotolerant fungal species can colonize the airways of patients with asthma, causing a range of clinical presentations grouped together as allergic fungal airway disease (AFAD) [1]. Exposure to fungal spores can trigger asthma exacerbations [2]. Current treatments for AFAD are not specific for asthma and are associated with limited efficacy or safety concerns [3].

Interleukin-33 (IL-33) levels are increased in lung epithelial cells and blood serum in patients with asthma [4]. The IL-33/suppressor of tumorigenicity 2 (ST2) pathway activates Th2-mediated and possibly non-Th2-mediated immune responses, leading to increased production of pro-inflammatory cytokines [4–6]. GSK3772847 (CNTO-7160), a human monoclonal antibody, inhibits IL-33 signaling by binding to the extracellular domain of the IL-33 receptor [7].

In a recently reported Phase I study, GSK3772847 was well tolerated in patients with mild asthma and target engagement was observed [7]. A Phase 2 Proof of Concept Study demonstrated a reduction in loss of asthma control with placebo in adults with moderate-to-severe asthma [8]. The current Phase IIa study evaluated the efficacy and safety of multiple doses of GSK3772847 compared with placebo in participants with moderate-to-severe asthma with AFAD receiving standard of care.

## Methods

### Study design

This was a 28-week randomized, multicenter, double-blind, sponsor-open, comparative study (see S1 File). The full study protocol is provided as S2 File. Participants were screened within 28 days prior to Week 0 (Visit 3). Participants that met the pre-defined randomization criteria were randomized (1:1) to GSK3772847 (10 mg/kg) or matching placebo intravenously administered at Weeks 0, 4, and 8 (visits 3, 5, and 6) in addition to standard of care. Randomization was stratified based on systemic anti-fungal treatment status at screening. Systemic steroids for asthma exacerbations were permitted but was not a screening/randomization criterion. Participants then attended a follow-up visit approximately 12 weeks after end of treatment (Visit 7). Blood eosinophils and fractional exhaled nitric oxide (FeNO) were measured at Weeks 0, 2, 4, 8, and 12. This study was conducted according to the principles of the Declaration of Helsinki, International Conference on Harmonization of Technical Requirements for Registration of Pharmaceuticals for Human Use (ICH) and Good Clinical Practice (GCP) and approved by the National Research Ethics Service Committee Yorkshire & The Humber–Leeds West, Jarrow, United Kingdom. All participants provided written informed consent. This study is registered at ClinicalTrials.gov (NCT03393806).

### Inclusion criteria

Eligible participants were aged ≥18 years with documented diagnosis of moderate-to-severe asthma (≥12 months) treated with inhaled corticosteroid and long-acting β2-agonist (≥4 months); pre-bronchodilator forced expiratory volume in 1 second 35–79% of predicted, FeNO ≥25 parts per billion, Asthma Control Questionnaire-5 score ≥1.5, and blood eosinophils ≥300 cells/μL at screening; evidence of AFAD (fungal sensitization to *Aspergillus fumigatus* [>0.35 KU/L] or *Penicillium chrysogenum* [>0.35 KU/L] measured by serum-specific immunoglobulin E) and a history of exacerbations (≥1 severe exacerbation in the previous 12 months); and no history of concurrent respiratory disease or recurrent or ongoing non-pulmonary infections.

### Study endpoints

Primary endpoints were change from baseline (Week 0) to Week 12 in blood eosinophils and FeNO. Key secondary endpoints were pharmacokinetics, serum levels of free soluble ST2 (sST2), and safety.

### Statistical analysis

Given the exploratory nature of the study, no formal sample size calculation was performed. Twenty participants per arm was estimated to provide an adequate level of precision for treatment differences for the primary endpoints.

Enrollment was terminated early due to recruitment issues and the unlikely unfeasibility of timely study completion. Consequently, all data were summarized, with no statistical analyses performed. Due to the small sample size at Week 12, there was inadequate estimation of the dispersion of the data, and the frequency distribution of the data was not normal. For this reason, the median and interquartile ranges are presented.

## Results

### Participant disposition and baseline demographics

**Fig 1:**
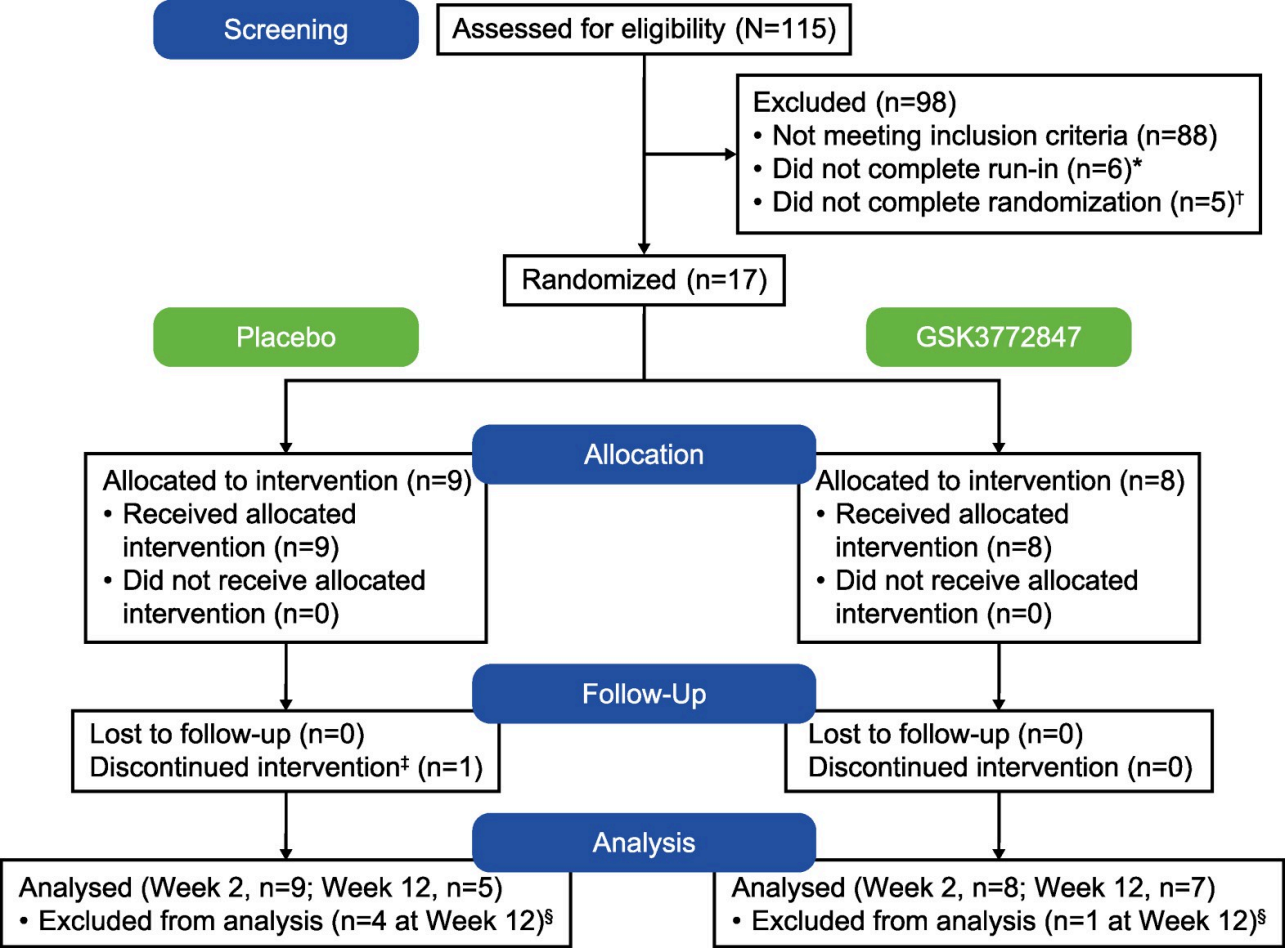
CONSORT flow diagram. *1 patient withdrew; 5 patients did not meet continuation criteria. †1 patient within this group was subsequently re-randomized and went on to complete the study. ^‡^Patient withdrew from the study (missed study visits). ^§^Patients were excluded if they had the Week 12 assessment outside of the treatment end date (+28 days).

Between 18 April 2018 and 6 January 2020, 115 participants were screened, of which 17 participants who met the inclusion criteria were randomized and included in efficacy and safety analyses: placebo, n = 9 (6 male); GSK3772847, n = 8 (6 male). The CONSORT flow diagram is shown in Fig 1. One participant in the placebo arm withdrew (missed study visits). Mean age was 56.9 years (standard deviation [SD]: 11.2), majority were white (88%), and median duration of asthma was 21.0 years (range: 4–63 years; Table 1).

**Table 1:** Baseline demographics and characteristics.

	Placebo (N = 9)	GSK3772847 (N = 8)
**Age in years, mean (SD)**	59.9 (9.57)	53.6 (12.59)
**Sex, n (%)**		
Male	6 (67%)	6 (75%)
Female	3 (33%)	2 (25%)
**BMI (kg/m2), mean (SD)**	26.89 (5.61)	29.55 (6.99)
**Race**		
White	9 (100)	6 (75)
Japanese/East Asian/South-East Asian	0	2 (25)
**Duration of asthma in years, mean (SD)**	28.6 (21.01)	23.9 (11.24)
**Onset of asthma in years, mean (SD)**	31.4 (22.52)	29.8 (17.74)

SD, standard deviation.

### Pharmacokinetics, target engagement, and efficacy

**Fig 2:**
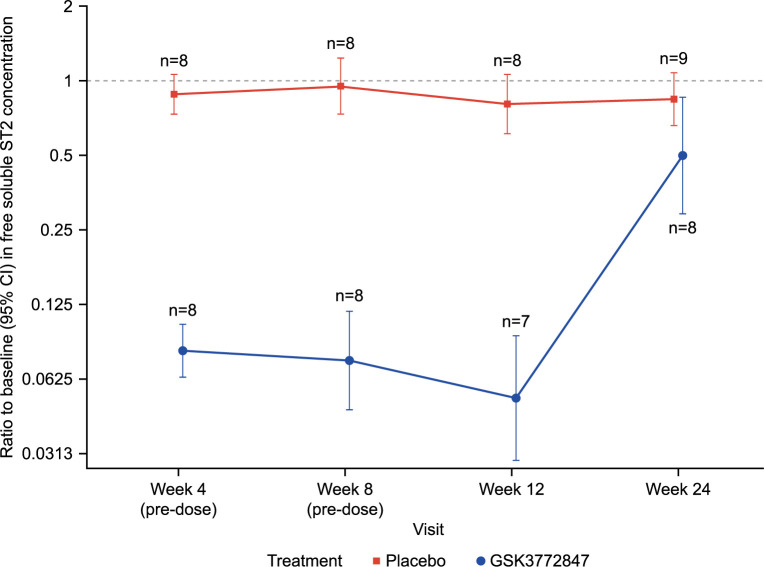
Ratio to baseline in free soluble ST2 levels*. *This figure includes on- and off-treatment data (on-treatment defined window is 28 days). CI, confidence interval; ST2, suppressor of tumorigenicity 2. The raw data for this figure are provided as S1 Table.

Mean C_max_ of GSK3772847 was 156.5 μg/mL (SD 74.1) and C_trough_ of 39.1–68.6 μg/mL. Free sST2 levels decreased in the GSK3772847 arm throughout the treatment period, whereas no reductions were observed with placebo (Fig 2; S1 Table). Post-treatment return to baseline in the GSK3772847 arm was consistent with a decline in GSK3772847 concentrations. Blood eosinophils decreased from baseline to Week 12 in the GSK3772847 arm (geometric mean decrease: 23.6%; median: 10.9%). In the placebo arm, there was a reduction in geometric mean (32.2%), but a slight increase in the median (9.7%). FeNO decreased from baseline to Week 12 in the GSK3772847 (geometric mean decrease: 49.6%; median: 45.6%) and placebo arms (geometric mean decrease: 41.5%; median: 37.2%) (Table 2).

**Table 2:** Change from baseline to Week 12 in eosinophils and FeNO.

		Week 0 (baseline)			Week 12		
		*n*	Geometric mean (%CV)	Median (Q1, Q3)	*n* *	Geometric mean change from baseline, %	Median change from baseline, % (Q1, Q3)
**Placebo (*N* = 9)**	**Eosinophils (10** ^**9**^ **/L)**	9	0.340 (69.2)	0.370 (0.230, 0.420)	5	–32.2	9.7 (–51.4, 30.4)
	**FeNO (ppb)**	9	45.8 (48.7)	44.0 (37.5, 51.0)	5	–41.5	–37.2 (–41.0, –37.2)
**GSK3772847 (*N* = 8)**	**Eosinophils (10** ^**9**^ **/L)**	8	0.442 (54.9)	0.585 (0.330, 0.614)	7	–23.6	–10.9 (–63.8, 3.8)
	**FeNO (ppb)**	8	52.3 (49.8)	51.0 (37.2, 66.8)	6	–49.6	–45.6 (–59.1, –35.9)

*Lower *n* values at Week 12 are due to some participants having the Week 12 assessment outside of the treatment end date (+28 days).

CV, coefficient of variation; FeNO, fractional exhaled nitric oxide; ppb, parts per billion; Min, minimum; Max, maximum; Q, quartile.

### Safety

Adverse events (AEs) were experienced by 3/8 (38%) participants with GSK3772847 and 7/9 (78%) participants with placebo, most commonly nasopharyngitis (GSK3772847, n = 1; placebo, n = 2) and headache (GSK3772847, n = 1; placebo, n = 2). No drug-related AEs or serious AEs were reported in the GSK3772847 arm. In the placebo arm, one drug-related AE was reported, and one participant experienced a non-fatal serious AE (prostate cancer). No deaths were reported. There were no clinically significant changes from baseline in electrocardiogram results or vital signs. One participant in each arm experienced an increase in QTcF values (>30 to ≤60 msec) post baseline.

## Discussion

This study was terminated early due to the high rate of screen failure, low enrollment, and unlikely feasibility of timely study completion. While other factors such as viruses, pollens, and chitin can induce or trigger IL-33 release, the choice to restrict the study to a single population (asthma) with a specific common inciting factor (fungal sensitization) associated with epithelial damage was made to eliminate any potential differences in the biological effect based on the inciting trigger [4, 9]. Consequently, as a result of the low subject numbers, interpretation of the data is limited.

GSK3772847 exposures were generally consistent with observations in previous clinical studies. Target engagement was confirmed via a significant reduction in free sST2 levels and an increase in total sST2, consistent with results from the previously reported Phase I study [7]. However, no differences were observed in blood eosinophils or FeNO between treatment arms at Week 12 in our study, which contrast with previous data showing positive results with other anti-IL-33 antibodies in the treatment of asthma [7, 8, 10]. In a phase II study, single-dose etokimab (ANB020) led to a decrease in blood eosinophil relative to baseline during the course of treatment [10]. In another Phase II study, monotherapy with REGN3500/SAR440340 (ST2 blocking mAb) significantly reduced loss of asthma control and improved lung function compared with placebo in adults with moderate-to-severe asthma [8]. These results support the clinical development of treatments targeting IL-33 or ST2 for patients with asthma. Unfortunately, treatment effects could not be detected in the present study given the small sample size.

No deaths occurred in this study and the safety observations are consistent with the known safety profile of GSK3772847.

Besides impacting sST2 levels, no conclusions can be drawn due to the early termination of the study and low population size. Nevertheless, it may be valuable to further evaluate the efficacy and safety of GSK3772847 in a larger population with moderate-to-severe asthma with AFAD.

## Supporting information

S1 File CONSORT checklist. (DOC)

S2 File Full study protocol. (PDF)

S1 Table Raw data for Fig 2: Ratio to baseline in free soluble ST2 levels. (DOCX)
