# Supplementary material for: Efficacy and safety of GSK3772847 in participants with moderate-to-severe asthma with allergic fungal airway disease: A phase IIa randomized, multicenter, double-blind, sponsor-open, comparative trial
Source: PLoS One. 2023 Feb 3;18(2):e0281205. doi: 10.1371/journal.pone.0281205 (PMC9897512; doi:10.1371/journal.pone.0281205)
Supplement: S2 File — (PDF) [file pone.0281205.s002.pdf]

# TITLE PAGE

**Protocol Title:** A double blind (sponsor open) placebo-controlled, stratified, parallel group study to evaluate the efficacy and safety of repeat doses of GSK3772847 in participants with moderate to severe asthma with allergic fungal airway disease (AFAD)

**Protocol Number**: 207972/02

**Short Title**: Repeat dose study of GSK3772847 in participants with moderate to severe asthma with allergic fungal airway disease (AFAD).

**Compound Number:** GSK3772847

**Sponsor Name and Legal Registered Address:**

GlaxoSmithKline Research & Development Limited
980 Great West Road
Brentford
Middlesex, TW8 9GS
UK

**Medical Monitor Name and Contact Information** can be found in the Study Reference Manual

**Regulatory Agency Identifying Number(s): EudraCT number: 2017-003544-20**

**Approval Date: 10-OCT-2018**

[redacted]

**SPONSOR SIGNATORY:**

PPD

PPD

Courtne PPD

Director, Clinical Development

**Date**

Respiratory Medicines Discovery and Development

GlaxoSmithKline

PPD

[redacted] [redacted]

# French Information Page

**Title of Protocol:** A double blind (sponsor open) placebo-controlled, stratified, parallel group study to evaluate the efficacy and safety of repeat doses of GSK3772847 in participants with moderate to severe asthma with allergic fungal airway disease (AFAD).

Protocol Reference: 207972
Effective Date of Protocol: 10 October 2018

Study Identifier:

<u>French Contacts:</u>

**GLAXOSMITHKLINE**
23, rue François JACOB
92500 RUEIL MALMAISON

Medical Project Leader:

Dr PPD
Tel : PPD

Study Team Leader:

PPD
Tel : PPD
Fax : PPD

Safety Contact:

PPD
Tel : PPD
Fax : PPD

Clinical Research Associate

**FRENCH SIGNATORY**

Jacques ROUX
Clinical Operations Director
GSK FRANCE

Date & Signature

PPD

[redacted] [redacted]

# French Coordinating Investigator Statement

**Title of Protocol:** A double blind (sponsor open) placebo-controlled, stratified, parallel group study to evaluate the efficacy and safety of repeat doses of GSK3772847 in participants with moderate to severe asthma with allergic fungal airway disease (AFAD).

Protocol Reference: 207972 / 02

Effective Date of Protocol: 10 October 2018

Study Identifier: 2017N331706_03

Compound Number: GSK3772847

I agree to act as the coordinating investigator for this clinical trial in France, according to the French law (n°2004-806 of 09 August 2004) "Article L1121-1 du Code de la Santé Publique".

French Coordinating Investigator Name: Professor Pascal CHANEZ

Date: PPD

French Coordinating Investigator Signature:

PPD

# PROTOCOL AMENDMENT SUMMARY OF CHANGES TABLE

| DOCUMENT HISTORY | |
|---|---|
| **Document** | **Date** |
| *Amendment 2-2017N331706_03* | *10-OCT-2018* |
| *Amendment 1 RUS-1-2017N331706_02* | *26-JUL-2018* |
| *Amendment 1-2017N331706_01* | *05-FEB-2018* |
| *Original Protocol-2017N331706_00* | *01-SEP-2017* |

**Amendment 2** 10-OCT-2018

**Overall Rationale for the Amendment:** To include participants with severe asthma with AFAD treated with low dose oral corticosteroid who still demonstrate a lack of complete control as demonstrated by ACQ-5, FeNO and blood eosinophil levels. Also, a few clarifications were included.

| Section # and Name | Description of Change | Brief Rationale |
|---|---|---|
| Section 6.1 Inclusion Criteria; Section 6.2 Table 2; Section 6.3 Run-in Exclusion Criteria; Section 6.5 Randomisation Exclusion Criteria; Section 7.7.1 Permitted Medications and Non-Drug Therapies; Section 7.7.2 Prohibited Medications and Non-drug Therapies | Additional therapy: with low dose oral corticosteroid (≤10 mg/day prednisolone or equivalent) is permissible.<br>High dose oral corticosteroid is defined as >10 mg/day prednisolone or equivalent. | It is not uncommon for severe asthma participants including those with allergic fungal airway disease to receive oral corticosteroids as maintenance treatment and still demonstrate a lack of complete asthma control. |
| Section 6.1 Inclusion Criteria; Section 6.7 Screening/Run-in/Randomisation Failures | Blood eosinophils of 250-299 cells/microliters at screening but with documented evidence of ≥300 cells /microliters within 5 months of screening will be accepted. | There is no well-accepted low cutoff point for eosinophilia between 150-300 cells/microliters. Blood eosinophils of 250 cells/microliters at screening with documented evidence of eosinophilia will be adequate to demonstrate efficacy and safety. |
| Section 9.1.2 Fractional Exhaled Nitric Oxide; Section 9.1.4 Spirometry | Participants should withhold use of their SABA and LABA, ICS and OCS medications prior to FeNO and Spirometry assessments. | Clarification to ensure that all participants perform key study assessments at the end of their SoC medication dosing intervals. |
| Section 9.8.1 Exploratory Biomarkers | Participants should withhold use of their SABA and LABA, ICS and OCS medications prior to visits with sputum assessments, similar to spirometry restrictions. | To ensure that baseline and end-of-treatment sputum samples are taken in similar conditions. |
| Section 2 Schedule of Activities; Section 9.8.2 Immunogenicity Assessments | Clarified that immunogenicity assessments should be performed pre dose. | Immunogenicity assessments should be done pre dose at all dosing visits. |
| Section 2 Schedule of Activities | Clarified that serum pregnancy test is required at screening. | WOCBP should only be included after a confirmed menstrual period and a negative highly sensitive serum pregnancy test. |
| Section 7.7.1 Permitted Medications and Non-Drug Therapies | Medications for rhinitis are disallowed 48 hours prior to ECG measurements | Reworded the sentence for clarification. |
| Section 7.7.2 Prohibited Medications and Non-drug Therapies | Methotrexate, troleandomycin, oral or parenteral gold, cyclosporin, azathioprine, cyclophosphamide, tacrolimus, mycophenolate mofetil, D-penicillamine are not permitted. | Clarification to match Table 2 list of Section 6.2 Exclusion Criteria. |

**TABLE OF CONTENTS**

**PAGE**

PROTOCOL AMENDMENT SUMMARY OF CHANGES TABLE....................................3

1. SYNOPSIS....................................8

2. SCHEDULE OF ACTIVITIES (SOA)....................................11

3. INTRODUCTION....................................17
   3.1. Study Rationale....................................17
   3.2. Background....................................17
   3.3. Benefit/Risk Assessment....................................19
      3.3.1. Risk Assessment....................................20
      3.3.2. Benefit Assessment....................................27
      3.3.3. Overall Benefit:Risk Conclusion....................................27

4. OBJECTIVES AND ENDPOINTS....................................28

5. STUDY DESIGN....................................30
   5.1. Overall Design....................................30
   5.2. Number of Participants....................................31
   5.3. Participant and Study Completion....................................31
   5.4. Scientific Rationale for Study Design....................................32
   5.5. Dose Justification....................................32

6. STUDY POPULATION....................................35
   6.1. Inclusion Criteria....................................35
   6.2. Exclusion Criteria....................................36
   6.3. Run-in Exclusion Criteria....................................40
   6.4. Randomisation Inclusion Criteria....................................40
   6.5. Randomisation Exclusion Criteria....................................40
   6.6. Lifestyle Restrictions....................................41
      6.6.1. Caffeine, Alcohol, and Tobacco....................................41
      6.6.2. Activity....................................41
   6.7. Screening/Run-in/Randomisation Failures....................................42

7. TREATMENTS....................................43
   7.1. Treatments Administered....................................43
      7.1.1. Medical Devices....................................44
      7.1.2. Rescue medication....................................44
   7.2. Dose Modification....................................44
   7.3. Method of Treatment Assignment....................................44
   7.4. Blinding....................................45
   7.5. Preparation/Handling/Storage/Accountability....................................46
      7.5.1. Accountability....................................46
      7.5.2. Preparation, Storage and Handling....................................46
   7.6. Treatment Compliance....................................47
   7.7. Concomitant Therapy....................................47
      7.7.1. Permitted Medications and Non-Drug Therapies....................................47
      7.7.2. Prohibited Medications and Non-drug Therapies....................................48

7.8. Treatment after the End of the Study ....... 49

8. DISCONTINUATION CRITERIA ....... 50
8.1. Discontinuation of Study Treatment ....... 50
8.1.1. Liver Chemistry Stopping Criteria ....... 51
8.1.2. QTc Stopping Criteria ....... 51
8.1.3. Rechallenge ....... 52
8.1.3.1. Study Treatment Restart or Rechallenge ....... 52
8.2. Withdrawal from the Study ....... 52
8.3. Lost to Follow Up ....... 53

9. STUDY ASSESSMENTS AND PROCEDURES ....... 54
9.1. Efficacy Assessments ....... 54
9.1.1. Blood Eosinophils ....... 54
9.1.2. Fractional Exhaled Nitric Oxide (FeNO) ....... 54
9.1.3. Questionnaires ....... 55
9.1.3.1. Asthma Control Questionnaire (ACQ-5) ....... 55
9.1.3.2. Asthma Quality of Life Questionnaire (AQLQ) ....... 55
9.1.4. Spirometry ....... 56
9.2. Adverse Events ....... 56
9.2.1. Time Period and Frequency for Collecting AE and SAE Information ....... 56
9.2.2. Method of Detecting AEs and SAEs ....... 57
9.2.3. Follow-up of AEs and SAEs ....... 57
9.2.4. Regulatory Reporting Requirements for SAEs ....... 57
9.2.5. Cardiovascular and Death Events ....... 57
9.2.6. Pregnancy ....... 58
9.3. Treatment of Overdose ....... 58
9.4. Safety Assessments ....... 58
9.4.1. Physical Examinations ....... 59
9.4.2. Vital Signs ....... 59
9.4.3. Electrocardiograms ....... 59
9.4.4. Continuous ambulatory ECG (Holter) ....... 59
9.4.5. Clinical Safety Laboratory Assessments ....... 60
9.4.6. Diary assessments ....... 60
9.5. Pharmacokinetics ....... 60
9.6. Pharmacodynamics ....... 61
9.7. Genetics ....... 61
9.8. Biomarkers ....... 61
9.8.1. Exploratory Biomarkers ....... 61
9.8.2. Immunogenicity Assessments ....... 61
9.9. Medical Resource Utilization and Health Economics ....... 62

10. STATISTICAL CONSIDERATIONS ....... 63
10.1. Sample Size Determination ....... 63
10.2. Populations for Analyses ....... 65
10.3. Statistical Analyses ....... 66
10.3.1. Efficacy Analyses ....... 66
10.3.2. Safety Analyses ....... 67
10.3.3. Pharmacokinetic Analyses ....... 67
10.3.4. Pharmacodynamic Analyses ....... 67
10.3.5. Other Analyses ....... 67

10.3.6. Interim Analyses ........ 68

11. REFERENCES ........ 69

12. APPENDICES ........ 71
12.1. Appendix 1: Abbreviations and Trademarks ........ 71
12.2. Appendix 2: Clinical Laboratory Tests ........ 74
12.3. Appendix 3: Study Governance Considerations ........ 77
12.4. Appendix 4: Adverse Events: Definitions and Procedures for Recording, Evaluating, Follow-up, and Reporting ........ 81
12.5. Appendix 5: Contraceptive Guidance and Collection of Pregnancy Information ........ 87
12.6. Appendix 6: Genetics ........ 90
12.7. Appendix 7: Liver Safety: Required Actions and Follow-up Assessments ........ 91
12.8. Appendix 8 : Protocol Amendment History ........ 94
12.9. Appendix 9 : French administrative considerations and specific requirements 98

# 1. SYNOPSIS

**Protocol Title:** A double blind (sponsor open) placebo-controlled, stratified, parallel group study to evaluate the efficacy and safety of repeat doses of GSK3772847 in participants with moderate to severe asthma with allergic fungal airway disease (AFAD).

**Short Title:** Repeat dose study of GSK3772847 in participants with moderate to severe asthma with allergic fungal airway disease (AFAD).

**Rationale:**

GSK3772847 (formerly CNTO 7160, which was in-licensed from Janssen) is a human immunoglobulin G2 sigma isotype (IgG2σ) antibody that binds Domain 1 of the cell-surface interleukin-33 receptor (IL-33R). Inhibition of IL-33 signalling via blockade of the IL-33 receptor (Suppressor of tumorigenicity 2 [ST2], also known as Interleukin-1 receptor like-1 [IL-1RL1]) presents a potential novel treatment for severe asthma as an add-on to standard of care. Agents targeting this mechanism could be expected to have effects on both type 2 (T2)-driven and non-T2-driven disease.

Allergic fungal airway disease (AFAD) is defined as Immunoglobulin E (IgE) sensitisation to thermotolerant filamentous fungi particularly *Aspergillus fumigatus*. Clinical data suggest increased IL-33 levels in bronchoalveolar (BAL) fluid and endobronchial biopsy at baseline disease of children with severe asthma with fungal sensitisation and in lung tissue samples from patients with allergic bronchopulmonary aspergillosis [In-house unpublished data]. Recent data reported an association between IgE sensitisation to colonising thermotolerant filamentous fungi and the presence of lung damage as demonstrated by high-resolution computed tomography (HRCT) scanning in moderate to severe asthma. This study aims to evaluate the effects of GSK3772847 in moderate to severe asthma participants with AFAD on top of standard of care.

**Objectives and Endpoints:**

| Objectives | Endpoints |
|---|---|
| **Primary** | |
| To evaluate the efficacy of 3 doses of GSK3772847 (administered every 4 weeks) compared with placebo in moderate to severe asthma participants with allergic fungal airway disease (AFAD) who are currently on Standard of Care (SoC) | • Change from baseline (Week 0) in blood eosinophils over time<br>• Change from baseline (Week 0) in fractional exhaled nitric oxide (FeNO) over time |
| **Secondary** | |
| To evaluate the serum pharmacokinetics (PK) of 3 doses of GSK3772847 (administered every 4 weeks) in moderate to severe asthma participants with AFAD | • Serum concentrations of GSK3772847 |
| To evaluate the pharmacodynamics (PD) of GSK3772847 in moderate to severe asthma participants with AFAD | • Serum levels of free and total soluble ST2 |

| Objectives | Endpoints |
|---|---|
| To evaluate the levels and specificity of any anti-drug antibodies formed following dosing with GSK3772847 | • Incidence and titres of serum anti-GSK3772847 antibodies post dosing |
| To evaluate the health status of moderate to severe asthma participants with AFAD currently on SoC, and who are treated with GSK3772847 compared with placebo-treated participants | • Change from baseline (Week 0) in Asthma Control Questionnaire -5 (ACQ-5) absolute score at Weeks 2, 4, 8 and 12<br>• Change from baseline (Week 0) in Asthma Quality of Life Questionnaire (AQLQ) total and domain scores at Weeks 2, 4, 8 and 12<br>• Proportion of responders to ACQ-5. A responder to ACQ-5 will be defined as a subject who has a decrease from baseline in ACQ-5 score of 0.5 or more.<br>• Proportion of responders to AQLQ. A responder to AQLQ will be defined as a subject who has an increase from baseline in AQLQ score of 0.5 or more. |
| To evaluate the effect on lung function of moderate to severe asthma participants with AFAD currently on SoC, treated with GSK3772847 compared with placebo | • Change from baseline (Week 0) in spirometry parameters over time including but not limited to pre-bronchodilator Forced expiratory volume in 1 second ($FEV_1$) |
| To evaluate the safety and tolerability of GSK3772847 compared with placebo in moderate to severe asthma participants with AFAD | Safety and tolerability parameters include:<br>• Treatment emergent adverse events (AE)<br>• Clinical Laboratory safety data<br>• Vital signs (blood pressure, heart rate)<br>• 12–Lead Electrocardiogram (ECG) monitoring<br>• 24-hour Holter monitoring |

**Overall Design:**

This is a randomised, multicentre, double blind (sponsor open), placebo-controlled, stratified, parallel group study evaluating 3 doses of 10 mg/kg GSK3772847 administered every 4 weeks versus placebo in addition to SoC. Participants who have been diagnosed as having moderate to severe asthma with allergic fungal airway disease shall be enrolled in the study. Moderate to severe asthmatics are defined as asthmatics who are well controlled with Step 3 or 4 treatment, respectively, based on Global Initiative for Asthma, 2017 Guidelines.

**Number of Participants:**

Approximately 46 participants with moderate to severe asthma will be randomised such that approximately 40 evaluable participants complete the study, where evaluable is

defined as subjects with at least one post-baseline measurement for both FeNO and blood eosinophils.

**Treatment Groups and Duration:**

Participants who meet the eligibility criteria at Screening (Visit 1) will enter a two-week Run-in period during which they will undergo one 24h Holter monitoring session and fulfil paper Diary requirements. Participants who meet the pre-defined randomisation criteria, will be randomised in a 1:1 ratio to one of the following treatment groups:

- 10 mg/kg GSK3772847 administered intravenously
- Matching placebo administered intravenously

The randomisation will be stratified based on whether a participant is taking anti-fungal medication or not, determined at screening.

Both treatments will be administered on Week 0 (Day 1), Week 4 and Week 8.

Each participant will:

- Be screened;
- Attend a run-in visit (Visit 2) and enter the run-in period, approximately 14 days before the first treatment visit;
- Receive a total of 3 doses, each one on separate study visits (Visits 3, 5 and 6); doses will be every 4 weeks starting at week 0;
- Attend treatment period study visits at week 2 (Visit 4) and week 12 (Visit 7) but not receive study treatment during these visits; and
- Have a follow-up visit (approximately 12 weeks after Visit 7).

Each participant will be involved in the study for approximately 28 weeks.

At specified visits, each participant will undergo the following procedures:

- Sputum inductions at baseline (Visit 2) and end of treatment period (Visit 7). The sputum inductions may each be repeated once: for baseline at visit 3 and for end of treatment period at an extra visit after Visit 7;
- 24 h Holter monitoring at Visits 2 and 3 only.

During treatment period study visits, the participant will:

- Have blood drawn, for PD and/or PK assessments;
- Undergo FeNO measurement and spirometry;
- Have vital signs measured; and
- Complete 2 Quality of Life (QoL) questionnaires.

## 2. SCHEDULE OF ACTIVITIES (SOA)

| **Procedure** | **Screening (±5days)** | **Run-in (±5days)** | **Treatment Period (Visit window ±3days)** | | | | **End of Treatment / EW (±3days)** | **FU (±3days)** | **Notes** |
|---|---|---|---|---|---|---|---|---|---|
| **Visit** | **V1** | **V2** | **V3** | **V4** | **V5** | **V6** | **V7** | **V8** | |
| **Week** | **-4** | **-2** | **0** | **2** | **4** | **8** | **12** | **24** | |
| **Day** | **-28** | **-14** | **1** | **15** | **29** | **57** | **85** | **169** | |
| Informed consent | X | | | | | | | | May be obtained prior to Screening. |
| Inclusion and exclusion criteria | X | X | X | | | | | | Recheck clinical status before 1st dose of study medication. |
| Demography | X | | | | | | | | |
| Full physical examination | X | | | | | | | | To include height and weight |
| Medical/medication/ drug/alcohol/smoking history | X | | | | | | | | Including smoking history, substance abuse, medical conditions and family history of premature cardiovascular disease; asthma disease duration and exacerbation history |
| Human immunodeficiency virus (HIV), Hepatitis B and C screening | X | | | | | | | | If test otherwise performed within 3 months prior to first dose of study treatment, testing at screening is not required |
| **Genetics** | | | | | | | | | |
| Informed consent for Pharmacogenetic blood sample | | X | | | | | | | |

| Procedure | Screening (±5days) | Run-in (±5days) | Treatment Period (Visit window ±3days) | | | | End of Treatment / EW (±3days) | FU (±3days) | Notes |
|---|---|---|---|---|---|---|---|---|---|
| **Visit** | **V1** | **V2** | **V3** | **V4** | **V5** | **V6** | **V7** | **V8** | |
| **Week** | **-4** | **-2** | **0** | **2** | **4** | **8** | **12** | **24** | |
| Pharmacogenetic (PGx) blood sample | | | X | | | | | | PGx sample may be drawn any time from Visit 3 onwards, pre-dose. Informed consent must be obtained before collecting a sample. |
| **Study Treatment and Questionnaires** | | | | | | | | | |
| Randomisation | | | X | | | | | | |
| Study treatment | | | X | | X | X | | | |
| Dispense diary card | | X | | | | | | | To be completed daily. |
| Diary card review | | | X | X | X | X | X | | |
| Collect diary card | | | | | | | X | | |
| Rescue medication dispensing | | X | X[1] | X[1] | X[1] | X[1] | | | 1. As needed |
| ACQ-5 | X | | X | X | X | X | X | | Test to be performed before all other assessments |
| AQLQ | | | X | X | X | X | X | | Test to be performed immediately after ACQ-5 |
| **Efficacy** | | | | | | | | | |
| Haematology (including eosinophil count) | X | | X[1] | X | X | X | X | | 1. Pre-dose |
| FeNO | X | | X | X | X | X | X | | Test to be performed pre-dose |
| Spirometry | X | | X | X | X | X | X | | Test to be performed pre-dose |

| Procedure | Screening (±5days) | Run-in (±5days) | Treatment Period (Visit window ±3days) | | | | End of Treatment / EW (±3days) | FU (±3days) | Notes |
|---|---|---|---|---|---|---|---|---|---|
| Visit | V1 | V2 | V3 | V4 | V5 | V6 | V7 | V8 | |
| Week | -4 | -2 | 0 | 2 | 4 | 8 | 12 | 24 | |
| Free and total sST2 (serum) | | | X[1] | X | X[2] | X[1] | X[3] | X[3] | 1. Pre and Post Dose<br>2. Pre Dose<br>3. Anytime (± 5 days)<br>Pre-dose samples: within 2 hours from the planned dosing time<br>Post-dose samples as soon as possible after end of infusion but must be taken within 4 hours. |
| Total & fungal specific IgE | X[1] | | X[2] | X | X | X | X | | 1. Fungal specific IgE only if no historical documented results available<br>2. Pre-dose |

| Procedure | Screening (±5days) | Run-in (±5days) | Treatment Period (Visit window ±3days) | | | | End of Treatment / EW (±3days) | FU (±3days) | Notes |
|---|---|---|---|---|---|---|---|---|---|
| **Visit** | **V1** | **V2** | **V3** | **V4** | **V5** | **V6** | **V7** | **V8** | |
| **Week** | **-4** | **-2** | **0** | **2** | **4** | **8** | **12** | **24** | |
| Induced sputum sample for biomarkers | | X | X[1,2] | | | | X[3] | | 1. If no viable sample is produced at V2, induction should be repeated pre dose at V3<br>2. If a participant does not produce a viable baseline sputum sample at visit 2 or at visit 3, the participant will not need to undergo a sputum induction at the end of the treatment period.<br>3. If no viable sample is produced, induction should be repeated after a minimum of 72 hrs but no later than 7 days after scheduled visit. |

| Procedure | Screening (±5days) | Run-in (±5days) | Treatment Period (Visit window ±3days) | | | | End of Treatment / EW (±3days) | FU (±3days) | Notes |
|---|---|---|---|---|---|---|---|---|---|
| **Visit** | **V1** | **V2** | **V3** | **V4** | **V5** | **V6** | **V7** | **V8** | |
| **Week** | **-4** | **-2** | **0** | **2** | **4** | **8** | **12** | **24** | |
| **Pharmacokinetics** | | | | | | | | | |
| Serum blood sample for PK | | | X[1] | X | X[2] | X[3] | X[4] | X[4] | 1. Post Dose<br>2. Pre Dose<br>3. Pre and Post Dose<br>4. Anytime (± 5 days)<br>Pre-dose samples: within 2 hours from the planned dosing time<br>Post-dose samples as soon as possible after end of infusion but must be taken within 4 hours. |
| **Safety** | | | | | | | | | |
| Laboratory assessments | X[1,2] | | X[1,3] | X[1] | X[1,3] | X[1] | X[1,3] | X[1] | 1. Clinical chemistry (includes liver chemistry)<br>2. Routine urinalysis at screening (Visit 1)<br>3. Cardiac markers<br>Note: haematology assessments in efficacy section |
| Serum blood sample for immunogenicity | | | X[1] | X | X[1] | X[1] | X | X | 1. Pre Dose |

| **Procedure** | **Screening (±5days)** | **Run-in (±5days)** | **Treatment Period (Visit window ±3days)** | | | | **End of Treatment / EW (±3days)** | **FU (±3days)** | **Notes** |
|---|---|---|---|---|---|---|---|---|---|
| **Visit** | **V1** | **V2** | **V3** | **V4** | **V5** | **V6** | **V7** | **V8** | |
| **Week** | **-4** | **-2** | **0** | **2** | **4** | **8** | **12** | **24** | |
| Urine or serum pregnancy test (WOCBP only) | X[1] | | X | | X | X | X | X | To be performed pre-dose during the treatment period<br>1. Serum pregnancy test required |
| 12-lead ECG | X | | X[1] | | X[1] | X[1] | X | | 1. Test to be performed pre-dose and post-dose within 30 mins after end of infusion. |
| 24 hour Holter | | X | X[1] | | | | | | Holter monitor needs to be returned to the clinic at end of 24 hour recording (i.e. the next day).<br>[1]Place the Holter 30-60mins prior to dosing |
| Vital signs | X | X | X[1] | | X[1] | X[1] | X | X | 1. Test to be performed prior to the 12-lead ECG (pre-dose and post dose) |
| AE review | | | ←================================→ | | | | | | |
| SAE review | X | X | ←================================→ | | | | | | At V1 and V2 collect only SAEs considered as related to study participation. |
| Concomitant medication review | X | X | ←================================→ | | | | | | |

EW: Early Withdrawal. The list of assessments listed in this column should be completed for an early withdrawal visit.

# 3. INTRODUCTION

GSK3772847 (formerly CNTO 7160, which was in-licensed from Janssen) is a human immunoglobulin G2 sigma isotype (IgG2σ) antibody that binds Domain 1 of the cell-surface interleukin-33 receptor (IL-33R). Inhibition of IL-33 signalling via blockade of the IL-33 receptor (Suppressor of tumorigenicity 2 [ST2], also known as interleukin-1 receptor like-1 [IL-1RL1]) presents a potential novel treatment for severe asthma as an add-on to standard of care. Agents targeting this mechanism could be expected to have effects on both type 2 (T2)-driven and non-T2-driven disease.

At the time of writing this protocol, a two-part, single and multiple ascending dose first time in human study has completed dosing (GlaxoSmithKline Document Number 2017N344518_00). The safety information from this study is included in the investigator brochure.

Another study, a Phase IIa, multicentre, randomised, placebo-controlled, double-blind, stratified, parallel group study in moderately severe asthmatics started in September 2017 and aims to complete in late 2018.

The present study is a Phase IIa study to provide supplemental efficacy, safety and tolerability, pharmacokinetic (PK) and pharmacodynamic (PD) profiles of GSK3772847 in patients with moderate to severe asthma with allergic fungal airway disease (AFAD).

## 3.1. Study Rationale

This study aims to evaluate the effects of GSK3772847 in moderate to severe asthma participants with AFAD on top of standard of care. Evidence suggests that in order to observe the maximum benefit of targeting the IL-33 signalling pathway with GSK3772847, participants would need to be in a state of pulmonary cellular damage. Clinical data suggest increased IL-33 levels in bronchoalveolar lavage (BAL) fluid and endobronchial biopsy at baseline disease of children with severe asthma with fungal sensitisation [Castanhinha, 2015]. Also, lung tissue samples from patients with allergic bronchopulmonary aspergillosis indicate a markedly increased IL-33 expression [In-house unpublished data]. Recent data reported an association between immunoglobulin E (IgE) sensitisation to colonising thermotolerant filamentous fungi and the presence of graded lung damage as demonstrated by high-resolution computed tomography (HRCT) scanning (such as bronchiectasis, bronchial wall thickness, air trapping, etc.) in moderate to severe asthma [Woolnough, 2016]. Therefore, the target population is moderate to severe asthmatics who have allergic fungal airway disease (defined as IgE sensitisation to thermotolerant filamentous fungi particularly *Aspergillus fumigatus*, including allergic bronchopulmonary aspergillosis [ABPA]) [Rick, 2016]. Moderate to severe asthmatics are defined as asthmatics who are well controlled with Step 3 or 4 treatment, respectively, based on Global Initiative for Asthma [GINA, 2017] Guidelines.

## 3.2. Background

Asthma is a serious public health problem affecting about 300 million individuals worldwide. Asthma is a chronic inflammatory disorder of the airways, defined by a

history of respiratory symptoms of variable length and intensity together with variable airflow limitation. These symptoms are often triggered by allergen or irritant exposure, exercise or viral respiratory infections [GINA, 2017]. When uncontrolled, asthma can place severe limitations on daily life, and is sometimes fatal.

Severe asthma represents approximately 5-10% of the asthma population and is associated with a greater frequency of asthma exacerbations, decreased health-related quality of life and greater symptom burden [Chung, 2014], [Aburuz, 2007, Moore, 2007). Fungal exposure has been associated with asthma severity [O'Driscoll, 2005]. Moderate to severe asthmatics who are sensitised to *Aspergillus fumigatus* have impaired lung function, as demonstrated by reduced forced expiratory volume in 1 second ($FEV_1$), more severe airway obstruction, and the requirement for higher doses of corticosteroids [Denning, 2006] [Menzies, 2011]. *Aspergillus fumigatus* (thermotolerant filamentous fungi) are able to colonise the airways and cause lung damage [Moss, 2014]. Susceptible hosts such as moderate to severe asthmatics with reduced mucocilliary clearance functions are unable to efficiently clear their respiratory epithelium of inhaled fungal spores. This leads to increased contact of fungal spores with the immune system, thereby facilitating fungal growth and mucosal colonisation [Tracy, 2016]. The resulting immune response in susceptible hosts is a deviation towards a Th2- driven immune response with increased production of Th2 cytokines, in part driven by IL-33 that is derived from the airway epithelium.

GSK3772847 binds to the extracellular domain of IL-33R and neutralizes IL-33-mediated IL-33R signalling. The IL-33R gene codes for both a soluble form (sST2) and a membrane-bound “long” form (ST2L or IL-33R). Soluble ST2 exists in the serum and is elevated in severe asthmatics during an exacerbation [Smithgall, 2008, Oshikawa, 2001].

IL-33R is expressed on a variety of immune and non-immune cells. IL-33 has been shown to be released after endothelial or epithelial cell damage during trauma, physicochemical / microbarometric stress or infection [Arshad, 2016]. The IL-33R pathway causes downstream production of Type 2 cytokines and contributes to Th2-mediated pathologies and allergic responses [Yagami, 2010, Smithgall, 2008], but has also been shown to promote Th1- and Th17-mediated responses [Arshad, 2016, Smithgall, 2008]. Inhibition of IL-33 signalling may result in down regulation of immune cell responses and therefore presents a potential novel treatment for severe asthma on top of standard of care [Arshad, 2016].

In a 3-month good laboratory practice (GLP) toxicology study, GSK3772847 was administered to cynomolgus monkeys as a weekly 15-minute intravenous (IV) infusion (20 or 100 mg/kg) and was found to be well-tolerated at both doses.

A Phase I randomised, double-blind, placebo-controlled, IV single ascending dose study in healthy participants and multiple ascending dose study in participants with asthma and participants with atopic dermatitis (Study CNTO7160ASH1001) has completed dosing. The final clinical study report is still pending. There are no efficacy data available to date.

More information about the non-clinical and clinical studies is available in the GSK3772847 Investigator's Brochure (IB) (GlaxoSmithKline (GSK) Document Number 2017N316832_00).

## 3.3. Benefit/Risk Assessment

More detailed information about the known and expected benefits and risks and reasonably expected adverse events of GSK3772847 may be found in the IB (GSK Document Number 2017N316832_00).

### 3.3.1. Risk Assessment

| Potential Risk of Clinical Significance | Summary of Data/Rationale for Risk | Mitigation Strategy |
|---|---|---|
| **Investigational Product (IP) GSK3772847** | | |
| **Cardiovascular (CV)**<br><br>There is evidence to suggest that the IL-33/ST2 pathway may be protective in the cardiovascular system. Components of the IL-33/ST2 pathway are expressed in a number of cellular components of the heart and blood vessels in rodents and human patients.<br><br>Increased circulating levels of soluble ST2 are markers of a poor prognosis in patients with hemodynamic stress (e.g. hemodynamic-hypertrophy, chamber dilation, fibrosis; ischemic-apoptosis and infarct volume). The effect was abolished in rodents with genetic knockout of ST2.<br><br>Atherosclerotic plaque development was significantly reduced in ApoE -/- mice given exogenous IL-33 while plaques were larger in mice treated with soluble ST2 (which binds and blocks IL-33). | **Non-clinical**:<br>No GSK3772847-related changes noted in (non-GLP) IV and subcutaneous (SC) 4 week monkey study at doses ≤100 mg/kg/week, or in the GLP 3 month IV repeat dose toxicity study at doses ≤100 mg/kg/week) or SC administration. However, it should be noted that the animals in toxicity studies are healthy and, therefore, are unlikely to detect the potential target related CV liability.<br><br>**Clinical**:<br>In Janssen study CNTO 7160ASH1001, several episodes of sinus tachycardia on telemetry were reported in a 20-year-old male healthy volunteer, between 1 and 9 hours post-dose (10 mg/kg), accompanied on one occasion by mild vertigo and malaise (no chest pain). Troponin I, N-terminal prohormone of brain natriuretic peptide (NT-proBNP) were normal pre dose and Day 5, also normal ECG and vital signs including temperature. The event was considered by the investigator to be likely related to investigational product. No specific cause was identified. Data on this event was reviewed by GSK (Internal Cardiac Safety Panel and Chief Medical Officer), | Exclude participants with existing clinically significant organic heart disease (e.g. Coronary artery disease [CAD], ACC/AHA Stage C/D, and New York Heart Association (NYHA) Class III/IV heart failure) and abnormal, clinically significant findings from 12-lead electrocardiogram (ECG) and 24-hour Holter monitoring (Section 6.2 and Section 6.3).<br>CV events will be monitored (including ECG and Holter monitoring) as specified in SoA (Section 2). All cardiac-related adverse events (AEs) will be reviewed by an internal safety review committee (iSRC, see Appendix 3). Protocol-defined stopping criteria are specified in Section 8.1. |

| Potential Risk of Clinical Significance | Summary of Data/Rationale for Risk | Mitigation Strategy |
|---|---|---|
| | and was not considered to impact further clinical development.<br>During 12 hours post-dose telemetry monitoring in Part 1 of Janssen study CNTO7160ASH1001, 6 out of 60 completed subjects were assessed to have abnormal findings by the investigator. The abnormalities were considered clinically significant and recorded as treatment emergent adverse events.<br><br>In Part 2 of the Janssen study CNTO 7160ASH1001, there were four reports of non-sustained ventricular tachycardia. Of these reports, one participant received placebo and two received GSK3772847 at 3 mg/kg and one received GSK3772847 at 10 mg/kg. The events were non-symptomatic, and a monomorphic pattern (i.e., not Torsades de pointes), which is a pattern thought not to be indicative of increased risk for sudden ventricular tachycardia and sudden death. Heart rate (HR) analysis did not identify any safety concern (no pattern of increased HR suggestive of an increase in sympathetic tone). All 4 participants had normal results from exercise test and echocardiogram. Dosing was continued as planned, and the dose escalated to 10 mg/kg. | |

| Potential Risk of Clinical Significance | Summary of Data/Rationale for Risk | Mitigation Strategy |
|---|---|---|
| **Increased risk of infections & immunosuppression**<br>Studies in mice indicate a potential role for IL-33 in infection control. IL-33 was shown to activate neutrophils in BALB/c mice subjected to cecal ligation and puncture thus preventing polymicrobial sepsis. Similarly, IL-33 is thought to stimulate neutrophil recruitment from the bone marrow to the periphery in response to fungal infection. Mice infected with flu virus and administered an IL-33 inhibitor exhibited a lower number of clusters of differentiated CD90+ and cluster of differentiated CD25+ inate lymphoid cells with consequent impaired lung function compared to phosphate buffered saline treated controls. IL-33 has also been shown to be produced in the helminth infected cecum of parasite infected mice and is shown to be important in expulsion of the parasite. | **Preclinical:**<br>No GSK3772847-related changes in clinical signs, white blood cell count or no microscopic changes (inflammatory cell infiltrates) in any tissues indicative of infection observed in monkey 4 week IV/SC or IV 3 month toxicity at doses ≤100 mg/kg/week.<br><br>**Clinical:**<br>Safety data from Janssen study CNTO7160ASH1001 Single Ascending Dose has shown the most frequent AEs reported as infections, including nasopharyngitis, rhinitis, gastroenteritis and upper respiratory tract infection. The frequency of these events was similar in GSK3772847 and placebo groups (18/45 [40%] of participants administered GSK3772847 versus 6/17 [35%] administered placebo.)[1]<br><br>Safety data from Part 2 of the Janssen study CNTO7160ASH1001 have shown the System-organ class with the most frequent AEs reported was 'Infections and infestations'. The number of asthma participants experiencing infections and infestations was similar between the group receiving GSK3772847 (9/18 [50%]) and the group receiving placebo (4/6 [66.7%]). The frequency of infection events in participants with | Participants with a known, pre-existing parasitic infestation within 6 months prior to Screening are excluded from participation in the study (Section 6.2).<br><br>Participants who develop an infection will be requested to seek medical advice, and subject to close monitoring.<br><br>European Union (EU) Regulatory guidance on development of asthma drugs request that agents that interact with the immune system should be investigated for their effect on the host response to infection and tumours. The incidence of infections will be monitored in clinical studies. Incidence of tumours and development of paradoxical immune responses (e.g. idiopathic thrombocytopenic purpura, autoimmune thyroiditis, multiple sclerosis-like syndrome) will be monitored in clinical trials and routinely as part of the post marketing pharmacovigilance process.<br><br>Exclude participants with ongoing or recurrent infections.<br><br>Close monitoring of infection AEs (including pneumonia). |

| Potential Risk of Clinical Significance | Summary of Data/Rationale for Risk | Mitigation Strategy |
|---|---|---|
| | atopic dermatitis was greater in participants in the GSK3772847 group compared with the placebo group (6/11 [54.5%] versus 1/4 [25%]). The most frequently-reported infection was nasopharyngitis. | |
| **Increased risk of hyper-sensitivity, anaphylaxis, cytokine release syndrome (CRS)**<br>Therapy with other monoclonal antibodies has been associated with hypersensitivity reactions which may vary in severity and time of onset. | **Nonclinical:**<br>Not observed in studies to date.<br><br>**Clinical:**<br>Not observed in Janssen study CNTO7160ASH1001 in healthy volunteers following single doses up to 10 mg/kg, and multiple doses in asthma and atopic dermatitis participants at doses up to 10 mg/kg (3 doses, once every two weeks over four weeks).<br>Based on in vitro cytokine release data and safety experience in Janssen study CNTO7160ASH1001 the risk of CRS is considered negligible. | If a hypersensitivity or anaphylactic reaction occurs, infusion should be discontinued immediately and appropriate therapy instituted. Agents to treat reactions should be available immediately. Stopping & continuation criteria will be included in protocols. Painkillers can be prescribed for pain at site of injection. Patients developing hypersensitivity, anaphylactic reactions or anaphylactic shock will be withdrawn from the study. All doses in this trial will be administered in the clinic. |
| **Possible interaction with live virus or bacterial vaccines**<br>As GSK3772847 is an immunomodulator, there is a possibility that the participant will not mount an adequate immune response to a vaccine or even cause the infection the vaccine should protect against.<br>Vaccination also drives a systemic immune response to the pathogen antigen that runs the risk of causing some immunomodulation of the | **Non-clinical:**<br>In the monkey 13 week toxicity study no GSK3772847-related changes in the T cell dependent B cell response (Immunoglobulin M [IgM] or Immunoglobulin G [IgG]) was observed at doses ≤100 mg/kg/week. This data is indicative that healthy monkeys were able to mount a response against the antigen challenge during GSK3772847 administration at doses where near complete inhibition of IL-33 was | In the study, participants should not be vaccinated with live or attenuated vaccines within 4 weeks prior to receiving IP or up to 6 months after dose administration of GSK3772847. However, vaccines containing killed bacteria or inactivated virus will be permitted. |

| Potential Risk of Clinical Significance | Summary of Data/Rationale for Risk | Mitigation Strategy |
|---|---|---|
| lung immune responses. This has been best studied in murine models where eosinophilic lung inflammation has been suppressed by systemic toll-like receptor activation. | anticipated.<br><br>Based on this data GSK3772847 is considered unlikely to blunt/inhibit the generation of a response to vaccinations.<br><br>**Clinical:**<br>Not observed in Janssen study CNTO7160ASH1001 in healthy volunteers following single doses up to 10 mg/kg, and multiple doses in asthma and atopic dermatitis patients at doses up to 10 mg/kg (3 doses, once every two weeks over four weeks). | |
| **Gastrointestinal disorders**<br><br>• Nausea<br>• Vomiting | **Clinical**<br>In Part 2 of Janssen study CNTO7160ASH1001, the incidence of gastrointestinal disorders was greater in participants in the GSK3772847 groups compared with the placebo groups: 6/18 (33.3%) participants in the asthma cohort and 3/11 (27.3) participants in the atopic dermatitis cohort as compared with 0 participants in either the asthma cohort or atopic dermatitis cohort placebo groups. Gastrointestinal disorders events included nausea (1/18 participants in the asthma cohort, 2/11 participants in the atopic dermatitis cohort), vomiting, diarrhoea. | The incidence and severity of nausea and vomiting will be monitored. |

| Potential Risk of Clinical Significance | Summary of Data/Rationale for Risk | Mitigation Strategy |
|---|---|---|
| **<u>Skin and subcutaneous tissue disorders</u>**<br><br>• Contact dermatitis | **<u>Clinical</u>**<br>In Part 2 of Janssen study CNTO7160ASH1001, the incidence of contact dermatitis was greater in participants in the GSK3772847 groups compared with the placebo groups. In the asthma cohort, the number of participants with contact dermatitis was 4/18 (22.2%) in the combined GSK3772847 groups versus 1/6 (16.7%) in the placebo group. In the atopic dermatitis, the number of participants with contact dermatitis was 3/11 (27.3%) in the combined GSK3772847 versus 0/4 (0%) in the placebo group. | The incidence and severity of contact dermatitis will be monitored. |

| Potential Risk of Clinical Significance | Summary of Data/Rationale for Risk | Mitigation Strategy |
|---|---|---|
| **Study Procedures** | | |
| Sputum Induction | Sputum production is stimulated by inhalation of increasing concentrations of saline solution. This inhalation may cause an increase in cough, which may lead to pain in the throat or chest as well as to breathlessness, due to irritation of the airways. | Participants will receive a bronchodilator (e.g. salbutamol) prior to the procedure to reduce discomfort. Lung function will be assessed at baseline (pre- and post-bronchodilator) and after each saline inhalation procedure. The procedure will be terminated if the $FEV_1$ drops below 20% of the post-bronchodilator value. If the $FEV_1$ falls by 10% of the post-bronchodilator value, the saline concentration will not be increased during the next inhalation period.<br><br>If the participant exhibits respiratory symptoms such as wheeze, shortness of breath or chest discomfort at any stage during the procedure, they may be given bronchodilator therapy as needed and the procedure terminated as appropriate. |

[1]Values have been updated from an incorrect entry in the IB and risk management benefit plan. Both documents will be updated with these correct values at the next scheduled update.

### 3.3.2. Benefit Assessment

Efficacy of GSK3772847 has not yet been demonstrated and there are no existent data from molecules with the same or similar mode of action. Taking part in this study may or may not improve a participant's health, and may or may not directly benefit a participant. This study will provide additional safety and efficacy information on GSK3772847.

Whilst the in vivo models of T2 asthma support a role for IL-33 pathway in eosinophilic asthma disease, it is clear that IL-33 plays a significant role in other types of immune responses and cell types including amplification of Th1 and Th17 responses in combination with other cytokines [Arshad, 2016; Smithgall, 2008]. Agents targeting this mechanism could be expected to have effects on both type 2 (T2)-driven and non-T2-driven disease.

All study participants will receive a rescue inhaler to use as needed for asthma symptom relief from Run-in to the end of the Treatment Period. Medical assessments are planned during the study to evaluate participants' health status. The assessments include physical examination, vital signs, ECG, Holter monitoring, and clinical laboratory evaluation including liver chemistry and blood chemistry panel at a number of clinic visits. Participants' health status will also be evaluated by ACQ-5 and AQLQ during the study. Participants' safety will also be assured by having criteria for withdrawal from study medication in case of loss of asthma control. The aim will be to retain participants in the study post withdrawal of study medication to follow-up for safety.

### 3.3.3. Overall Benefit:Risk Conclusion

Taking into account measures to minimize the risk to the participants participating in the study, the balance of anticipated benefits and apparent risks associated with GSK3772847 continues to be acceptable. There is an opportunity to determine if there is a new drug that can be developed for patients with severe asthma who may benefit from the broad spectrum effects hypothesized for GSK3772847.

# 4. OBJECTIVES AND ENDPOINTS

| Objectives | Endpoints |
|---|---|
| **Primary** | |
| To evaluate the efficacy of 3 doses of GSK3772847 (administered every 4 weeks) compared with placebo in moderate to severe asthma participants with allergic fungal airway disease (AFAD) who are currently on Standard of Care (SoC) | • Change from baseline (Week 0) in blood eosinophils over time<br>• Change from baseline (Week 0) in fractional exhaled nitric oxide (FeNO) over time |
| **Secondary** | |
| To evaluate the serum pharmacokinetics (PK) of 3 doses of GSK3772847 (administered every 4 weeks) in moderate to severe asthma participants with AFAD | • Serum concentrations of GSK3772847 |
| To evaluate the pharmacodynamics (PD) of GSK3772847 in moderate to severe asthma participants with AFAD | • Serum levels of free and total soluble suppressor of tumorigenicity 2 (ST2) |
| To evaluate the levels and specificity of any anti-drug antibodies formed following dosing with GSK3772847 | • Incidence and titres of serum anti-GSK3772847 antibodies post dosing |
| To evaluate the health status of moderate to severe asthma participants with AFAD currently on SoC, and who are treated with GSK3772847 compared with placebo-treated participants | • Change from baseline (Week 0) in Asthma Control Questionnaire -5 (ACQ-5) absolute score at Weeks 2, 4, 8 and 12<br>• Change from baseline (Week 0) in Asthma Quality of Life Questionnaire (AQLQ) total and domain scores at Weeks 2, 4, 8 and 12<br>• Proportion of responders to ACQ-5. A responder to ACQ-5 will be defined as a subject who has a decrease from baseline in ACQ-5 score of 0.5 or more.<br>• Proportion of responders to AQLQ. A responder to AQLQ will be defined as a subject who has an increase from baseline in AQLQ score of 0.5 or more. |
| To evaluate the effect on lung function of moderate to severe asthma participants with AFAD currently on SoC, treated with GSK3772847 compared with placebo | • Change from baseline (Week 0) in spirometry parameters over time including but not limited to pre-bronchodilator Forced expiratory volume in 1 second ($FEV_1$) |
| To evaluate the safety and tolerability of GSK3772847 compared with placebo in moderate to severe asthma participants with AFAD | Safety and tolerability parameters include:<br>• Treatment emergent adverse events (AE)<br>• Clinical Laboratory safety data<br>• Vital signs (blood pressure, heart rate)<br>• 12–Lead Electrocardiogram (ECG) monitoring<br>• 24-hour Holter monitoring |

| Objectives | Endpoints |
|---|---|
| **Exploratory** | |
| To evaluate changes in exploratory biomarkers in the blood of moderate to severe asthma participants with AFAD who have been treated with GSK3772847 compared with placebo | • Change over time in levels of serum total Immunoglobulin E (IgE), fungal-specific IgE |
| To evaluate changes in IL-33 related and disease biology biomarkers in the sputum of moderate to severe asthma participants with AFAD who have been treated with GSK3772847 compared with placebo | • Difference from placebo in levels of sputum biomarkers including but not limited to interleukin (IL)-33, IL-13, IL-4, IL-5 and TNF-$\alpha$<br>• Difference from placebo including but not limited to levels of sputum eosinophils |

# 5. STUDY DESIGN

## 5.1. Overall Design

This is a randomised, multicentre, double blind (sponsor open), placebo-controlled, stratified, parallel group study evaluating 3 doses of 10 mg/kg GSK3772847 administered every 4 weeks versus placebo in addition to SoC. Participants who have been diagnosed as having moderate to severe asthma with allergic fungal airway disease shall be enrolled in the study.

**Figure 1 Study Design Overview**

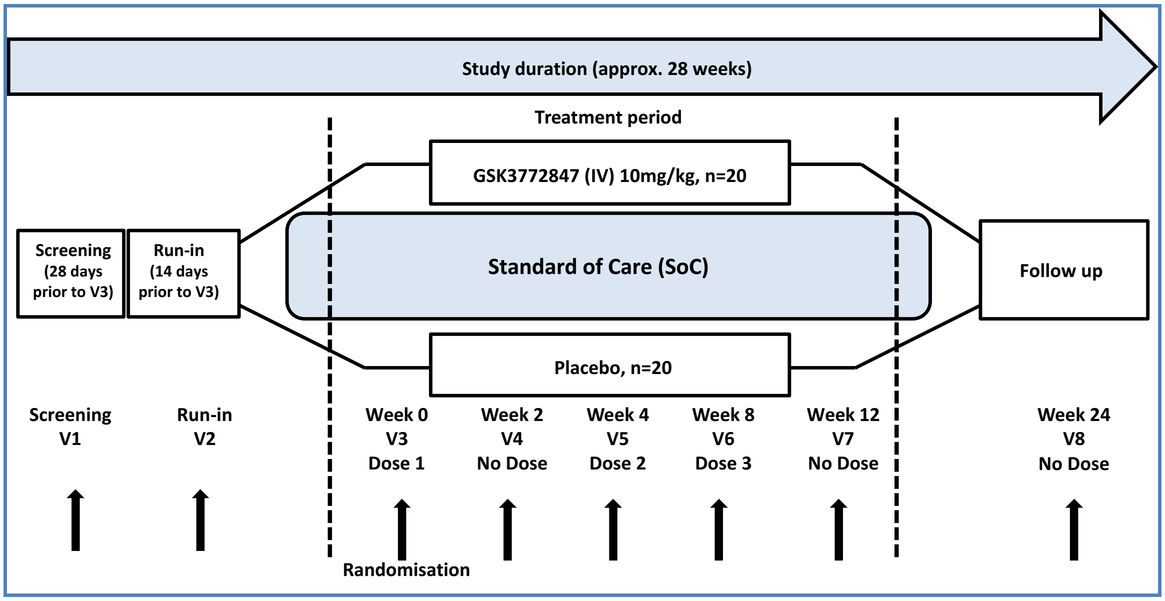


Participants who meet the eligibility criteria at Screening (Visit 1) will enter a two-week Run-in period during which they will undergo one 24h Holter monitoring session and fulfil paper Diary requirements. Participants who meet the pre-defined randomisation criteria (Section 6.4), will be randomised in a 1:1 ratio to one of the following treatment groups:

- 10 mg/kg GSK3772847 administered intravenously
- Matching placebo administered intravenously

The randomisation will be stratified based on whether a participant is taking anti-fungal medication or not, determined at screening.

Both treatments will be administered on Week 0 (Day 1), Week 4 and Week 8.

Each participant will:

- Be screened;

- Attend a run-in visit (Visit 2) and enter the run-in period, approximately 14 days before the first treatment visit;
- Receive a total of 3 doses, each one on separate study visits (Visits 3, 5 and 6); doses will be every 4 weeks starting at Week 0;
- Attend treatment period study visits at Week 2 (Visit 4) and Week 12 (Visit 7) but not receive study treatment during these visits; and
- Have a follow-up visit (approximately 12 weeks after Visit 7).

Each participant will be involved in the study for approximately 28 weeks.

At specified visits, each participant will undergo the following procedures:

- Sputum inductions at baseline (Visit 2) and end of treatment period (Visit 7). The sputum inductions may each be repeated once: for baseline at Visit 3 and for end of treatment period at an extra visit after Visit 7;
- 24 h Holter monitoring at Visits 2 and 3 only.

During treatment period study visits, the participant will:

- Have blood drawn, for PD and/or PK assessments;
- Undergo FeNO measurement and spirometry;
- Have vital signs measured; and
- Complete 2 Quality of Life (QoL) questionnaires.

For participants who discontinue IP early, but have not withdrawn consent to participate in the study, an early withdrawal (EW) visit will be performed 4 weeks after the last dose of blinded study treatment. These participants should continue in the study and complete all assessments at the remaining protocol-defined visits until their EW visit, and complete the follow-up visit assessments.

## 5.2. Number of Participants

Approximately 46 participants with moderate to severe asthma will be randomised such that approximately 40 evaluable participants complete the study, where evaluable is defined as subjects with at least one post-baseline measurement for both FeNO and blood eosinophils.

If participants prematurely discontinue the study, additional replacement participants may be recruited and assigned to the same treatment (from Visit 1), at the discretion of the Sponsor in consultation with the investigator.

## 5.3. Participant and Study Completion

A participant is considered to have completed the study if he/she has completed all phases of the study including screening, run-in, the randomised treatment phase, and the follow-up visit.

The end of the study is defined as the date of the last visit (Visit 8) of the last participant in the study.

## 5.4. Scientific Rationale for Study Design

This study will use a randomised, multi-centre, double blind (sponsor open), placebo-controlled, parallel-group design. This is a well-established design to evaluate the efficacy, safety, PK and PD profile of an investigational medicinal product.

Use of a placebo arm is considered justified as all patients will be continuing on standard of care treatments and will also allow the absolute effect of GSK3772847 to be assessed.

The study will be sponsor open to allow selected sponsor study team members to be unblinded in order to perform interim analysis of in-stream data.

The dosing duration of 8 weeks is supported by pre-clinical study data. Dosing frequency of GSK3772847 every 4 weeks with endpoints assessments scheduled 4 weeks post final dose were determined by the available target engagement pharmacodynamic findings.

Participants will be followed up for an additional 12 weeks before a final safety evaluation. This follow up period will ensure that sufficient PK samples are collected to characterise the pharmacokinetics, pharmacodynamics, and anti-drug antibody responses in this patient population.

## 5.5. Dose Justification

The dosing regimen of 10 mg/kg IV at Week 0 then Weeks 4 and 8 was selected based on the observed evidence of target suppression following single doses in healthy participants (CNTO7160ASH1001). In summary, administration of a single 10 mg/kg dose led to significant (>95%) suppression of serum free sST2 and sustained elevations of total sST2 up to at least 28 days after dosing. Therefore, the selected regimen should deliver significant target suppression throughout the treatment period, including at trough, and allow determination of the impact of targeting this pathway on the primary endpoint (measured over 0-12 weeks).

Simulations of exposure were generated using a preliminary Michaelis Menten (MM) population PK model using the single dose data from 0.03-10 mg/kg up to 28 days. Safety margins were estimated by comparing the mean clinical exposures (predicted or observed) against the mean observations in the 3-month GLP toxicology study in cynomolgus monkeys (T-2013-007). Area under the curve (AUC) margins were calculated by comparing the predicted clinical exposures from 0-28 weeks (end of study) to the observed exposure from 0-92 days (13 weeks) when the main study animals were removed from study. Predicted exposures throughout the study and follow up period are significantly lower than those observed in study T-2013-007 as shown in Figure 2.

The anticipated exposure margins of the dosing regimen of 10 mg/kg IV at Week 0 then Weeks 4, and 8 over the 3-month GLP toxicology study in cynomolgus monkeys (T-2013-007) are summarised in Figure 2.

**Figure 2 Predicted clinical exposures at 10mg/kg at weeks 0, 4, and 8 using a preliminary MM population PK model against observed exposures in study CNTO7160ASH1001 (part 1 single dose) and observed exposures at the No Observed Adverse Effect Level (100 mg/kg weekly) in the 3-month GLP toxicology study in cynomolgus monkeys (T-2013-007)**

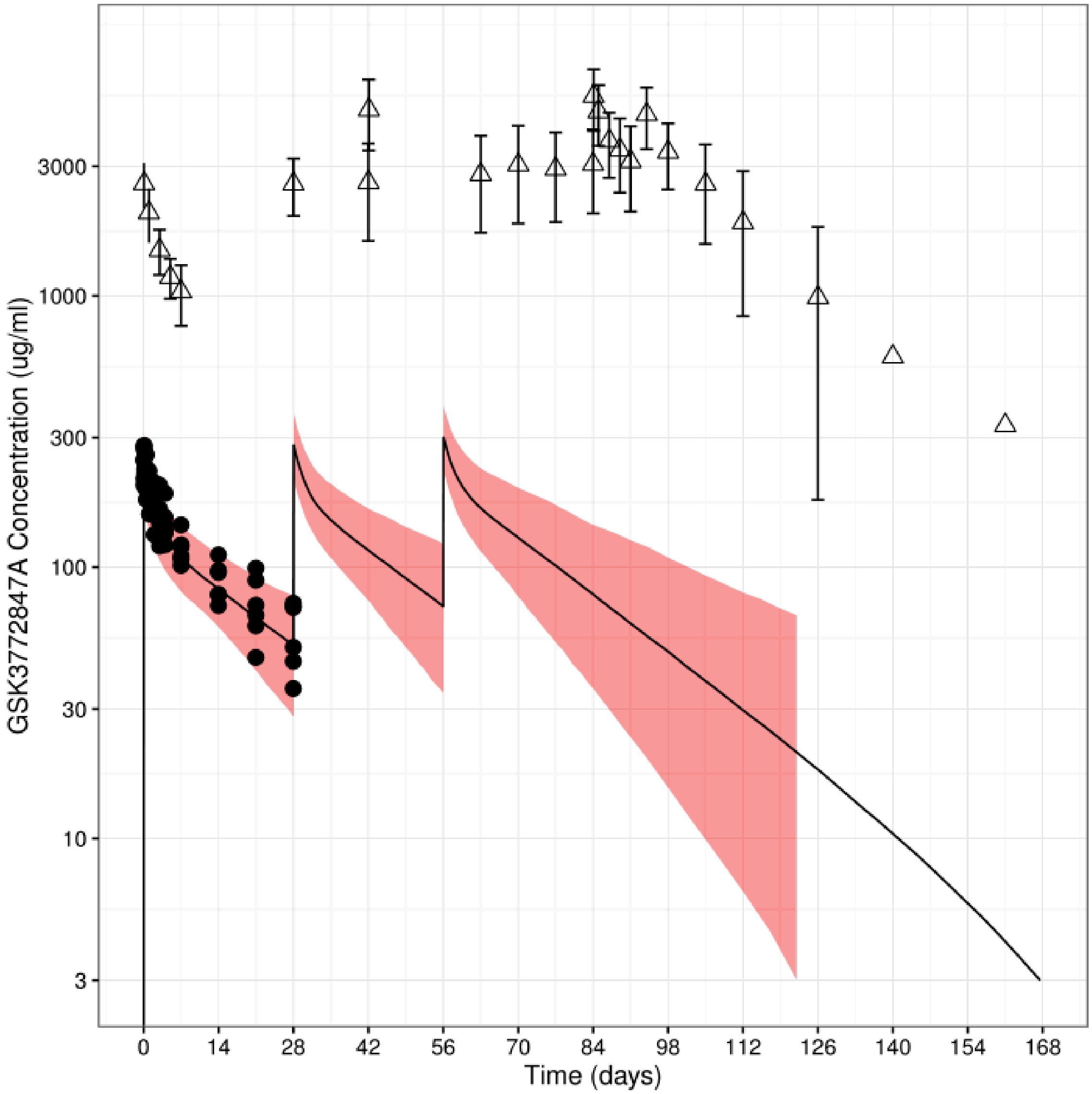


Open Triangles: Observed mean and standard deviation of exposures through main study and recovery phase (post week 13) in toxicology study T-2013-007.
Solid circles: Observed clinical exposures in part 1 of study CNTO7160ASH1001.
Solid line and shade region: median and 95% prediction interval for clinical exposures using a preliminary MM population PK model.

**Table 1 Predicted clinical exposures and safety margins for study 207972 following dosing of 10 mg/kg at weeks 0, 4, and 8.**

| Day 1 mean $C_{max}$ (µg/mL) | Exposure margin[a] | Week 8 mean $C_{max}$ (µg/mL) | Exposure margin[a] | 0-28 weeks mean AUC (µg.day/mL) | Exposure margin[b] |
|---|---|---|---|---|---|
| 245.3[c] | 10.5 | 304.5[d] | 17.9 | 12545.3[d] | 24.4 |

a. Margins calculated against mean maximum serum concentration ($C_{max}$) on day 1 and day 84 (last dose) in study T-2013-007 (2592.55 and 5444.07 µg/mL respectively).
b. Margins calculated based on AUC (0-92 (13 weeks)) estimated using compartmental modelling of mean exposures in study T-2013-007 (306900 ug.day/mL).
c. Mean observed exposures in study CNTO7160ASH1001.
d. Predicted exposures using preliminary MM model

As an additional approach to comparing exposure margins the AUC(τ) at steady state at 10 mg/kg every 4 weeks was estimated based on the preliminary population PK model. At this regimen, the contribution of the non-linear elimination is minimal and so AUC(inf) can be estimated as Dose/Clearance. Clearance was estimated at 2.38 mL/kg/day, therefore, mean predicted AUC(τ) at steady state is 4202 µg.day/mL.

The mean AUC(day85-92) at the no observed adverse effect level (NOAEL) (100 mg/kg, IV, weekly) in cynomolgus monkeys was 27408 µg.day/mL (T-2013-007). In order to correct for weekly dosing in the cynomolgus and dosing every 4 weeks in this study, this figure was multiplied by 4 and results in an estimated exposure margin of 26.

# 6. STUDY POPULATION

Prospective approval of protocol deviations to recruitment and enrolment criteria, also known as protocol waivers or exemptions, is not permitted.

## 6.1. Inclusion Criteria

Participants are eligible to be included in the study only if all of the following criteria apply:

**Age**

1. Participant must be at least 18 years of age inclusive, at the time of signing the informed consent.

**Type of Participant and Disease Characteristics**

2. Documented history of physician diagnosed moderate or severe asthma for ≥12 months based on [GINA, 2017] Guidelines and treated with inhaled corticosteroid (ICS) and long-acting beta-2-agonist (LABA) for at least 4 months (≥500 μg/day fluticasone propionate or equivalent as defined in the [GINA, 2017] guidelines). Additional therapy: with low dose oral corticosteroid (≤10 mg/day prednisolone or equivalent), with a leukotriene receptor antagonist (LTRA), with anticholinergics, with anti-fungal medication is permissible.
3. Pre-bronchodilator $FEV_1$ 35-79% of predicted value for participant inclusive
4. FeNO ≥25ppb at Screening (Visit 1)
5. ACQ-5 score ≥1.5 at Screening (Visit 1)
6. Blood eosinophils ≥300 cells/microliter at Screening (Visit 1). Participants with blood eosinophils of 250-299 cells/microliters (inclusive) at screening but with documented evidence of ≥300 cells /microliters within 5 months of screening will be eligible for the study.
7. Evidence of allergic fungal airway disease:

   Fungal sensitisation to at least one of the following fungi: *Aspergillus fumigatus*, *Penicillium chrysogenum (notatum)* at screening measured by serum-specific IgE test. Documented fungal sensitisation either by serum-specific IgE or skin prick test is also acceptable.

   A history of exacerbations (at least 1 severe exacerbation (defined as requiring a minimum of 3 days of high-dose oral corticosteroids [>10 mg/day prednisolone, or equivalent] for asthma symptoms) in the previous 12 months.

**Weight**

8. Body weight within 50-150 kg (inclusive).

**Sex**

9. Male and female

**a. Female participants:**

A female participant is eligible to participate if she is not pregnant (see Appendix 5), not breastfeeding, and at least one of the following conditions applies:

i. Not a woman of childbearing potential (WOCBP) as defined in Appendix 5

OR

ii. A WOCBP who agrees to follow the contraceptive guidance in Appendix 5 during the treatment period and for the duration of the follow-up period until the last visit at week 24 (or 16 weeks after the last dose, which is equivalent to 5.5 half-lives).

**Informed Consent**

10. Capable of giving signed informed consent as described in Appendix 3 which includes compliance with the requirements and restrictions listed in the informed consent form (ICF) and in this protocol.

## 6.2. Exclusion Criteria

Participants are excluded from the study if any of the following criteria apply:

**Medical Conditions**

1. Historical diagnosis of cystic fibrosis
2. Concurrent respiratory diseases: Presence of a known pre-existing, clinically important respiratory conditions (e.g. pneumonia, pneumothorax, atelectasis segmental or larger, pulmonary fibrotic disease, bronchopulmonary dysplasia, chronic bronchitis, emphysema, chronic obstructive pulmonary disease, or other respiratory abnormalities) other than asthma or AFAD
3. Has a history of chronic or recurrent non-pulmonary infectious disease or ongoing non-pulmonary infection including, but not limited to, chronic renal infection, chronic chest infection, recurrent urinary tract infection (eg, recurrent pyelonephritis, chronic non-remitting cystitis), or open, draining skin wound or an ulcer
4. Serious infection within 8 weeks of enrolment, including, but not limited to hepatitis, pneumonia, sepsis, or pyelonephritis; or has been hospitalised for an infection; or has been treated with IV antibiotics for an infection, within 8 weeks prior to the first administration of study drug
5. Evidence of poorly controlled chronic medical conditions other than asthma, e.g., patients with known, pre-existing, clinically significant endocrine, autoimmune, metabolic, neurological, renal, cardiovascular, gastrointestinal, hepatic, and haematological or any other system abnormalities that are uncontrolled with standard treatment.
6. Cardiovascular disease: Clinically significant organic heart disease (e.g. CAD, NYHA Class III/IV heart failure).
7. Participants with a diagnosis of malignancy or in the process of investigation for a malignancy. Participants with carcinoma that have not been in complete remission for

at least 5 years. Participants who have had carcinoma in situ of the cervix, squamous cell carcinoma and basal cell carcinoma of the skin would not be excluded based on the 5 year waiting period if the patient has been considered cured by treatment..

8. Eosinophilic diseases: Other conditions that could lead to elevated eosinophils such as hypereosinophilic syndromes. Participants with a known, pre-existing parasitic infestation within 6 months prior to Screening (Visit 1).

**Prior/Concomitant Therapy**

9. Use of the medications listed in Table 2 is not permitted within the defined time intervals prior to Screening (Visit 1) and throughout the study. Potential participants should not be washed out of their medication solely for the purpose of enrolling in the trial.

**Table 2 Prohibited Medications**

| Medication | Time interval prior to Screening |
|---|---|
| Investigational drug | 30 days or 5-half-lives (whichever is longer) |
| Biologic agents (such as monoclonal antibodies including marketed drugs) | 130 days or 5 half-lives whichever is longer |
| Live or attenuated vaccines | 2 weeks |
| Experimental anti-inflammatory drugs (nonbiologics) | 3 months |
| Oral corticosteroids (>10 mg/day prednisolone or equivalent) | ≥2 weeks as short-term treatment of asthma exacerbation<br>>4 weeks as SoC |
| Corticosteroids intramuscular, long acting depot | 3 months |
| Immunomodulatory/suppressive agents (e.g. Methotrexate, troleandomycin, oral or parenteral gold, cyclosporin, azathioprine, cyclophosphamide, tacrolimus, mycophenolate mofetil, D-penicillamine) | 3 months |
| Theophylline | 3 months |
| Chemotherapy and radiotherapy | 12 months |

**Diagnostic assessments**

10. Presence of hepatitis B surface antigen (HBsAg), positive hepatitis C antibody test result at screening or within 3 months prior to first dose of study treatment.

11. A known immunodeficiency such as human immunodeficiency virus infection.

12. Hypersensitivity: significant allergies to humanised monoclonal antibodies or biologic or to any components of the formulation used in this study.

13. Clinically significant multiple or severe drug allergies, intolerance to topical corticosteroids, or severe post-treatment hypersensitivity reactions (including, but

not limited to, erythema multiforme major, linear immunoglobulin a dermatosis, toxic epidermal necrolysis, and exfoliative dermatitis).

14. Clinically significant abnormality on 12-lead ECG assessment at screening (Visit 1). Site investigators will be provided with ECG over-read conducted by a centralised independent cardiologist, to assist in evaluation of participant eligibility. For this study, an abnormal and clinically significant ECG that would preclude a participant from entering the trial is defined as a 12-lead tracing that is interpreted as, but not limited to, any of the findings in Table 3.

**Table 3 Abnormal and clinically significant ECG findings**

- Sinus bradycardia <45 beats per minute (bpm)

**Note: Sinus bradycardia <45bpm should be confirmed by two additional readings at least 5 minutes apart.*

- Sinus tachycardia ≥110bpm

**Note: Sinus tachycardia ≥110 should be confirmed by two additional readings at least 5 minutes apart.*

- Multifocal atrial tachycardia (wandering atrial pacemaker with rate >100bpm)
- Evidence of Mobitz II second degree or third degree atrioventricular (AV) block
- Pathological Q waves (defined as wide [>0.04 seconds] and deep [>0.4mV (4mm with 10mm/mV setting)] or >25% of the height of the corresponding R wave, providing the R wave was >0.5mV [5mm with 10mm/mV setting], appearing in at least two contiguous leads

**Note: prior evidence (i.e., ECG obtained at least 12 months prior) of pathological Q waves that are unchanged are not exclusionary; and the investigator will determine if the participant is precluded from entering the study.*

- Evidence of ventricular ectopic couplets, bigeminy, trigeminy or multifocal premature ventricular complexes
- For participants without complete right bundle branch block: QT interval corrected for heart rate by Fridericia's formula (QTc[F]) ≥450 msec or an ECG that is unsuitable for QT measurements (e.g., poor defined termination of the T wave)
- For participants with complete right bundle branch block: QTc(F) ≥480 msec or an ECG that is unsuitable for QT measurements (e.g., poor defined termination of the T wave)

**Note: All potentially exclusionary QT measurements should be confirmed by two additional readings at least 5 minutes apart.*

- ST-T wave abnormalities (excluding non-specific ST-T wave abnormalities)

**Note: prior evidence (i.e., ECG obtained at least 12 months prior) of ST-T waves that are unchanged are not exclusionary and the investigator will determine if the participant is precluded from entering the study.*

- Clinically significant conduction abnormalities (e.g., Wolff-Parkinson-White syndrome or bifascicular block defined as complete left bundle branch block or complete right bundle branch block with concomitant left fascicular block)
- Clinically significant arrhythmias (e.g., atrial fibrillation with rapid ventricular response, ventricular tachycardia)

**Other Exclusions**

15. Smoking history: current smokers or former smokers with a smoking history ≥10 pack years (pack years = number of cigarettes smoked per day / 20 * number of years smoked). Former smokers are defined by ≥6 months abstention.

16. History of alcohol or illegal substance abuse within 2 years prior to Screening (Visit1).
17. Participants at risk of non-compliance, or unable to comply with the study procedures. Participants who are unable to follow study instructions such as visit schedule and paper diary completion. Participants who have known evidence of lack of adherence to controller medication and/or ability to follow physician's recommendations. Any infirmity, disability, or geographic location that would limit compliance for scheduled visits.

## 6.3. Run-in Exclusion Criteria

Participants meeting any of the following criteria should not enter the run-in period at Visit 2:

1. Clinically significant and abnormal laboratory finding at Screening (Visit 1): Evidence of clinically significant abnormal laboratory tests during screening which are still abnormal upon repeat analysis and are not believed to be due to disease(s) present. Each Investigator will use his/her own discretion in determining the clinical significance of the abnormality.
2. Liver function at Screening (Visit 1)
    a. Alanine Aminotransferase (ALT) >2x upper limit of normal (ULN) and bilirubin >1.5xULN (isolated bilirubin >1.5xULN is acceptable if bilirubin is fractionated and direct bilirubin >35 %).
    b. Current or chronic history of liver disease, or known hepatic or biliary abnormalities (with the exception of Gilbert's syndrome or asymptomatic gallstones.
3. Asthma exacerbation: Participants with ongoing severe asthma exacerbation at the time of Visit 2 (defined as requiring a minimum of 3 days of high-dose oral corticosteroids [>10 mg/day prednisolone or equivalent] for asthma symptoms).
4. Positive pregnancy test at Run-in if test is done at Visit 2.
5. Ongoing infection(s) requiring systemic antibiotics

## 6.4. Randomisation Inclusion Criteria

At the end of the Run-in period (Visit 3), study participants must fulfil the following additional criteria in order to be randomised into the study and enter the treatment period:

1. ACQ-5 score ≥1.5 at Visit3.
2. FeNO ≥25ppb at Baseline (Visit 3)

## 6.5. Randomisation Exclusion Criteria

Participants meeting any of the following criteria must not be randomised to double-blind study medication at Visit 3:

1. 24-Hour Holter Monitoring: An abnormal and significant finding from 24-hour Holter monitoring at Run-in (Visit 2). Investigators will be provided with Holter reviews conducted by an independent cardiologist to assist in evaluation of subject eligibility. Specific findings that preclude subject eligibility are listed in Table 3. The

study investigator will determine the medical significance of any Holter abnormalities not listed in Table 3.

2. Asthma exacerbation: Participants with ongoing severe asthma exacerbation at the time of Visit 3 (defined as requiring a minimum of 3 days of high-dose oral corticosteroids [>10 mg/day prednisolone or equivalent] for asthma symptoms).
3. Severe airflow obstruction: a pre-bronchodilator $FEV_1$ <35 % predicted of normal value at Visit 3. Predicted values will be based upon GLI [Quanjer, 2012] equations for spirometry reference values.
4. Positive pregnancy test at Visit 3
5. Ongoing infection(s) requiring systemic antibiotics.

## 6.6. Lifestyle Restrictions

### 6.6.1. Caffeine, Alcohol, and Tobacco

- During each dosing session, participants will abstain from ingesting caffeine- or xanthine-containing products (eg, coffee, tea, cola drinks, and chocolate) for 8 hours before the start of dosing until after collection of the final PK and/or pharmacodynamic sample.
- During each dosing session, participants will abstain from alcohol for 24 hours before the start of dosing until after collection of the final PK and/or pharmacodynamic sample.
- Use of tobacco or e-cigarette products will not be allowed from screening until after the final follow-up visit.

### 6.6.2. Activity

- Participants will abstain from strenuous exercise for 24 hours before each blood collection for clinical laboratory tests. Participants may participate in light recreational activities during studies (eg, watching television, reading).

## 6.7. Screening/Run-in/Randomisation Failures

A participant will be assigned a participant number at the time the informed consent is signed at Visit 1.

For the purposes of this study, participants who consent to participate in the clinical study but do not subsequently enter the study or receive study treatment will be defined as follows:

- **Screening failures**: those participants that complete at least one Screening (Visit 1) procedure but do not enter the run-in period.
- **Run-in failures**: those participants that complete at least one Visit 2 procedure but do not enter the double-blind study treatment period.
- **Randomisation failures**: those participants that complete at least one Visit 3 procedure but do not receive study treatment.

Any participant who completes the run-in period and then meets the randomisation criteria and receives the double-blind study treatment at Visit 3 is considered to have entered the treatment period.

A minimal set of screen failure information is required to ensure transparent reporting of screen failure participants to meet the Consolidated Standards of Reporting Trials publishing requirements and to respond to queries from regulatory authorities. Minimal information includes demography, screen failure details, eligibility criteria, and any serious adverse events (SAEs).

Rescreening is only allowed in exceptional circumstances of technical errors or at the Investigator's discretion in consultation with GSK if a participant experiences an asthma exacerbation either at run-in or randomisation. Rescreened participants will be assigned a new participant number. Rescreening of subjects who screen-failed based on inclusion criterion 6 prior to Amendment 02 implementation may be rescreened after consultation with GSK.

# 7. TREATMENTS

Study treatment is defined as any investigational treatment(s), marketed product(s), placebo, or medical device(s) intended to be administered to a study participant according to the study protocol.

## 7.1. Treatments Administered

| **Study Treatment Name:** | GSK3772847 | Placebo |
|---|---|---|
| **Dosage formulation:** | 50mg/mL GSK377284, 15 mM Sodium Phosphate, 8.5% [w/v] Sucrose, and 0.04% [w/v] Polysorbate 20, pH 7.3. | |
| **Unit dose strength(s)/Dosage level(s):** | 10 mg/kg | Commercially sourced sterile normal saline |
| **Route of Administration** | IV infusion | IV infusion |
| **Dosing instructions:** | GSK3772847 for injection will require further reconstitution and dilution at the study site prior to administration:<br>dilution between 10 and 30 mg/mL may be accomplished by using commercially sourced sterile normal saline | |
| **Packaging and Labeling** | Study Treatment will be provided as 100mg/vial, white to yellow, uniform lyophilized cake in a 5ml clear glass vial with 20mm closure sealed by red metal and yellow overseal.<br>Each container will be labelled as required per country requirement. | Commercially sourced sterile normal saline |

### 7.1.1. Medical Devices

- Other medical devices (not manufactured by or for GSK) provided for use in this study are 24h Holter monitor.
- Instructions for the 24h Holter monitor use are provided in the Holter monitor manual.

### 7.1.2. Rescue medication

A rescue inhaler for as needed use throughout the study will be dispensed starting at Visit 2. At the Investigator's discretion, more than one rescue inhalers may be dispensed at any one time. Short acting β-2 agonist (SABA) will be sourced from local commercial stock. The contents of the label will be in accordance with all applicable regulatory requirements. The investigator should attempt to match the participant's current medication or provide a suitable alternative. The use of rescue medications is allowable at any time during the study following randomisation. The date of rescue medication administration must be recorded on the rescue medication worksheet/diary card.

## 7.2. Dose Modification

There are no dose modifications planned for this protocol.

## 7.3. Method of Treatment Assignment

Participants will be assigned to study treatment in accordance with the randomisation schedule. The randomisation code will be generated by GSK using a validated computerised system. Participants will be randomised using an interactive web response system (IWRS). The study will use central-based randomisation to allocate treatments. Once a randomisation number is assigned to a participant, it cannot be reassigned to any other participant in the study.

Following the 2-week Run-in period and satisfaction of all eligibility criteria, participants will be randomised 1:1 to one of the following double-blind (sponsor open) treatments for the duration of the Treatment Period:

- GSK3772847 (10 mg/kg) administered intravenously
- Placebo administered intravenously

The duration of the Treatment Period for each participant is 12 weeks. Study treatment will be dispensed at the study visits summarized in the SoA (Section 2). Returned study treatment should not be re-dispensed to the participants.

Each Investigator will be provided with sufficient supplies to conduct the trial. Additional treatment kits will be supplied as needed to the sites. Details of how to use the IWRS system to randomise participants and manage study treatment supplies (including dispensing) is provided in the IWRS manual and the SRM.

## 7.4. Blinding

This will be a double blind (sponsor open) study and the following will apply:

- All study staff involved in clinical assessments (which includes the investigator, sub-investigators, other site staff), and the participant will be blinded to the treatment allocated to individual participants. Selected sponsor study team members (and delegates if programming activities are outsourced) will be unblinded to perform the interim analysis. This may include the study statistician, study programmer (and delegates) and study pharmacokineticist; however, only the statistician and programmer (and delegates) will have access to individual participant level data. Access to unblinded data will be kept to the minimum set of individuals required to implement any interim analyses, but may include GSK management/review committees if alterations to the study conduct are required. Details of who were unblinded to what data and when will be included in the clinical study report.
- The IWRS will be programmed with blind-breaking instructions. In case of an emergency, the investigator has the sole responsibility for determining if unblinding of a participant's treatment assignment is warranted. Participant safety must always be the first consideration in making such a determination. If the investigator decides that unblinding is warranted, the investigator should make every effort to contact GSK prior to unblinding a participant's treatment assignment unless this could delay emergency treatment of the participant. If a participant's treatment assignment is unblinded GSK must be notified within 24 hours after breaking the blind. The date and reason that the blind was broken must be recorded in the source documentation and Case Report Form (CRF), as applicable.
- In the event of unblinding by the investigator or treating physician the Medical monitor/GSK team should be contacted to determine whether subject withdrawal is required. Should a participant's treatment assignment be unblinded and the Medical monitor/GSK team determine that the participant must be withdrawn from study treatment, the participant must be followed-up as per protocol until the completion of the early withdrawal (EW) assessments (see SoA in Section 2).
- A participant whose treatment assignment is inadvertently unblinded (either to investigative staff or the participant themselves) will be permitted to remain in the study, although the accidental unblinding will be recorded as a protocol deviation and hence the participant will be subject to review as to their inclusion in analyses.

Participants will be randomised in a [1:1] ratio to receive study treatment. Investigators will remain blinded to each participant's assigned study treatment throughout the course of the study. In order to maintain this blind, an otherwise uninvolved 3rd party (such as an unblinded pharmacist or other appropriately licensed and authorised personnel) will be responsible for the reconstitution and dispensation of all study treatment and will endeavour to ensure that there are no differences in time taken to dispense following randomisation.

Unblinded monitors and in the event of a Quality Assurance audit, the auditor(s) will be allowed access to un-blinded study treatment records at the site(s) to verify that randomisation/dispensing has been done accurately.

GSK's Global Clinical Safety and Pharmacovigilance staff may unblind the treatment assignment for any participant with an SAE. If the SAE requires that an expedited regulatory report be sent to one or more regulatory agencies, a copy of the report, identifying the participant's treatment assignment, may be sent to investigators in accordance with local regulations and/or GSK policy.

## 7.5. Preparation/Handling/Storage/Accountability

### 7.5.1. Accountability

1. The investigator or designee must confirm appropriate temperature conditions have been maintained during transit for all study treatment received and any discrepancies are reported and resolved before use of the study treatment.
2. Only participants enrolled in the study may receive study treatment and only authorised site staff may supply or administer study treatment. All study treatments must be stored in a secure, environmentally controlled, and monitored (manual or automated) area in accordance with the labeled storage conditions with access limited to the investigator and authorised site staff.
3. The investigator, institution, or the head of the medical institution (where applicable) is responsible for study treatment accountability, reconciliation, and record maintenance (ie, receipt, reconciliation, and final disposition records).
4. Further guidance and information for the final disposition of unused study treatment are provided in the Study Reference Manual.

### 7.5.2. Preparation, Storage and Handling

GSK3772847 is provided as a white to yellow, uniform lyophilized cake in a 5ml clear glass vial with 20mm closure sealed by red metal and yellow overseal. Each vial contains 100 mg of a lyophilised GSK3772847. When reconstituted with 2.0 mL of water for injection, the final concentration of GSK3772847 is 50 mg/mL. Excipients include: sucrose, sodium phosphate buffer, and polysorbate 20 at a pH of 7.3. Vials contain no preservatives and thus are for single use. Vials must be stored 2° – 8°C, protected from light. Protection from light during preparation and administration is not required. Full details on specific 2° to 8°C storage temperature conditions, preparation and administration including requirements for filtration are provided in the unblinded site staff reference manual or study reference manual.

Commercially available sterile normal saline will be used for dilution of study agent and will also serve as placebo for this study. Use of study agent sterile normal saline as placebo for injection provides an adequate comparator to broadly assess safety in early clinical development.

GSK3772847 must be prepared by an unblinded pharmacist or other appropriately licensed and authorised personnel and administered according to each participant's body

weight at Screening (Visit 1). A different site staff member, who will be blinded to the treatment assignment, will administer the study agent. Aseptic procedures must be used during preparation and administration of the study agent. Diluted GSK3772847 at volumes of 50 mL are to be administered by IV infusion over a period of at least 30 minutes using an in-line 0.22 micron filter. At least 30 mL of commercially available sterile normal saline will be used to flush diluted drug from the administration set to ensure full study agent administration.

Unblinded site staff will be responsible for receipt, storage, reconstitution, and labelling, and accountability of investigational product.

GSK3772847 should be inspected visually for particulate matter and discoloration prior to administration whenever solution and container permit. If visibly opaque particles, discoloration, or other foreign particles are observed, the solution should not be used.

Detailed instructions for storage conditions, dose preparation, and administration will be provided in the unblinded site staff reference manual or study reference manual. Required storage conditions and expiration date are indicated on the label.

Under normal conditions of handling and administration, study treatment is not expected to pose significant safety risks to site staff. Take adequate precautions to avoid direct eye or skin contact and the generation of aerosols or mists. In the case of unintentional occupational exposure notify the monitor, Medical Monitor and/or GSK study contact.

A Material Safety Data Sheet/equivalent document describing occupational hazards and recommended handling precautions either will be provided to the investigator, where this is required by local laws, or is available upon request from GSK.

## 7.6. Treatment Compliance

- GSK3772847 or placebo will be intravenously administered to participants at the site. Administration will be documented in the source documents and reported in the electronic case report form (eCRF).

## 7.7. Concomitant Therapy

Any medication or vaccine (including over-the-counter or prescription medicines, vitamins, and/or herbal supplements) that the participant is taking at the time of enrolment or during the study must be recorded along with:

- reason for use
- dates of administration including start and end dates
- dosage information including dose and frequency

The Medical Monitor should be contacted if there are any questions regarding concomitant or prior therapy.

### 7.7.1. Permitted Medications and Non-Drug Therapies

The following medications are allowed during the study:

- ICS/LABA, SABA, LTRA and anticholinergics as defined in the inclusion criteria (Section 6.1 criterion 2), used in a consistent dosage and frequency from screening and throughout the study
- Low dose oral corticosteroids (≤10 mg prednisolone per day) are permitted as standard of care. This should have been started a minimum of 4 weeks prior to screening and should be maintained at that dose until the end of the treatment period (V7).
- Anti-fungal medications (oral) are permitted if treatment was started a minimum of 1 month from screening and must be used in a consistent dosage and frequency from screening and throughout the treatment period. If discontinued recently, a washout of 3 months prior to screening is required. Ad hoc use during the treatment period is not permitted.
- Medications for rhinitis (e.g., intranasal corticosteroids, antihistamines [including ocular and intranasal], cromolyn, nedocromil, nasal decongestants) are permitted but they are disallowed 48 hours prior to ECG measurements.
- Azithromicin used in a consistent dosage and frequency from screening and throughout the study.
- Antibiotics for short term treatment of acute infections. Long term treatment with topical or ophthalmic antibiotics are permitted.
- Decongestants: Participants may take decongestants during the study, but these are disallowed for 24 hours prior to ECG measurements.
- Topical and ophthalmic corticosteroids
- Paracetamol or Acetaminophen, at doses of ≤ 3 grams/day
- Hormone replacement therapy (HRT) or hormonal contraception

A participant who requires systemic corticosteroid for the treatment of a severe asthma exacerbation after randomisation will be permitted to continue the study. The Investigator, in consultation with the medical monitor, will determine if discontinuation of study treatment is required (Section 8).

Any medication, herbal therapy or vitamin which is not listed as prohibited in Section 7.7.2 is permitted at the discretion of the investigator.

### 7.7.2. Prohibited Medications and Non-drug Therapies

Use of the medications listed below is not permitted at any time during the study. Refer to Table 2 for the minimum time interval since discontinuation prior to screening if medications were used by participants before screening.

- Biologics, e.g. Mepolizumab and Omalizumab.
- Potent CYP3A4 inhibitors, (e.g., ritonavir, ketoconazole, etc.)
- Anticonvulsants (barbiturates, hydantoins, and carbamazepine).
- Polycyclic antidepressants.
- Beta-adrenergic blocking agents.
- Phenothiazines.

- Monoamine oxidase inhibitors.
- Live or attenuated vaccines (and up to 6 months after the last dose of blinded study treatment).
- Experimental anti-inflammatory drugs (non-biologics).
- Intramuscular and long acting depot corticosteroids.
- Methotrexate, troleandomycin, oral or parenteral gold, cyclosporin, azathioprine, cyclophosphamide, tacrolimus, mycophenolate mofetil, D-penicillamine.
- Theophylline.
- Chemotherapy and radiotherapy.

All concomitant medications may be reviewed by the Medical Monitor and it will be up to the discretion of the Investigator and if necessary the Medical Monitor, whether the medication can be continued and/or the participant can take part in the study.

## 7.8. Treatment after the End of the Study

Participants will not receive any additional treatment from GSK after completion of the study or withdrawal of IP because other treatment options are available.

The Investigator is responsible for ensuring that consideration has been given to the post-study care of the participant’s medical condition.

# 8. DISCONTINUATION CRITERIA

## 8.1. Discontinuation of Study Treatment

Participants that permanently stop study treatment are encouraged to remain in the study. Participants have the right to discontinue study treatment before the end of the study. A participant may also be asked to discontinue study treatment at the Investigator's discretion.

Participants who withdraw from study treatment prematurely (for any reason) should, where possible, continue to be followed-up until the completion of the Safety Follow-up assessments:

- For participants who discontinue IP early, but have not withdrawn consent to participate in the study, an EW visit will be performed 4 weeks after the last dose of blinded study treatment. These participants should continue in the study and complete all assessments at the remaining protocol-defined visits until their EW visit. Participants should continue regular treatment for their asthma, as determined by the investigator. The follow-up visits will then be performed 12 weeks after the EW visit for safety assessments.
- Participants who discontinue IP early and withdraw consent to participate in the study should complete as many assessments planned for the EW visit as possible.

If this is not possible, the Investigator must encourage the participant to participate in as much of the study as they are willing (or able) to.

A participant may be withdrawn from study treatment at any time. A reason for premature discontinuation of study treatment (e.g., AE, protocol deviation, Investigator discretion, consent withdrawn etc.) must be captured in the eCRF.

A participant must be withdrawn from study treatment if any of the following stopping criteria are met at any time during the study:

1. Liver Chemistry: Meets any of the protocol-defined liver chemistry stopping criteria.
2. QT interval corrected for heart rate (QTc): Meets any of the protocol-defined stopping criteria.
3. Pregnancy: Positive pregnancy test.
4. A clinically significant asthma exacerbation (requiring hospitalisation).
5. Abnormal Holter of Mobitz II AVB, complete AVB, sustained or non-sustained ventricular tachycardia (VT), paroxysmal supraventricular tachycardia (PSVT), new onset atrial fibrillation/flutter will be a withdrawal/stopping criterion. These findings on ECG (baseline [V2], V3) or findings of myocardial ischemia will also result in withdrawal/stopping.
6. Hypersensitivity or anaphylactic reaction

### 8.1.1. Liver Chemistry Stopping Criteria

**Liver chemistry stopping and increased monitoring criteria** have been designed to assure participant safety and evaluate liver event etiology.

Discontinuation of study treatment for abnormal liver tests should be considered by the investigator when a participant meets one of the conditions outlined in the algorithm or if the investigator believes that it is in the best interest of the participant.

**Phase II Liver Chemistry Stopping and Increased Monitoring Algorithm**

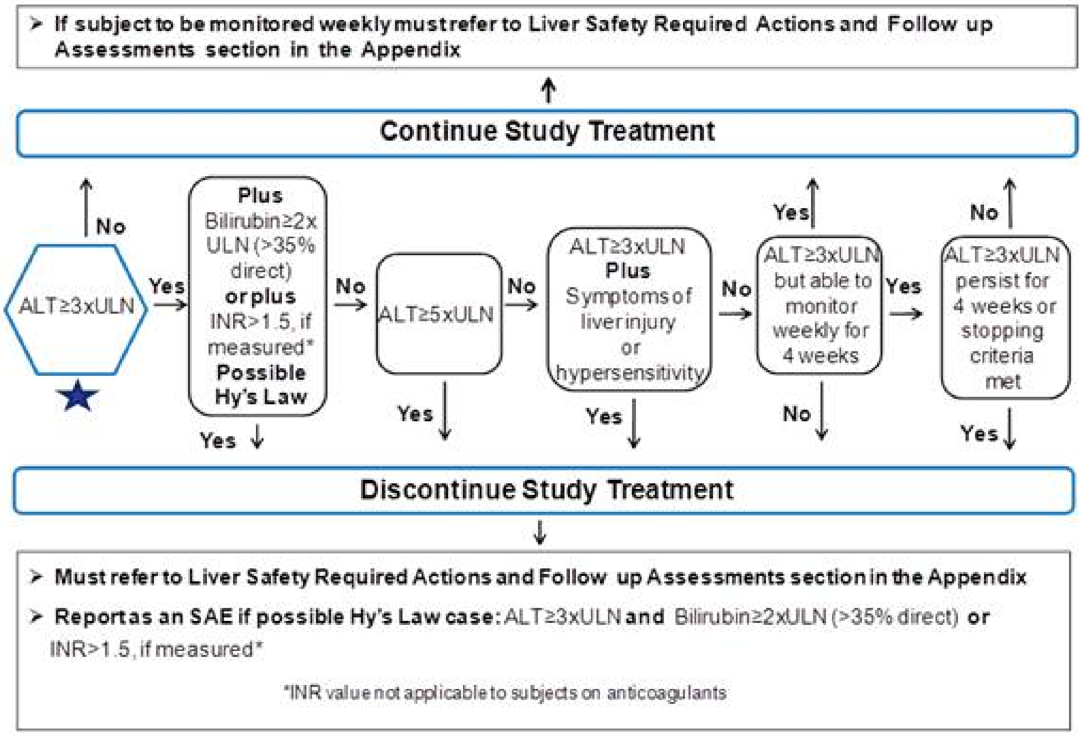


Liver Safety Required Actions and Follow up Assessments Section can be found in Appendix 7.

### 8.1.2. QTc Stopping Criteria

- The QTc is the QT interval corrected for heart rate according to Bazett's formula (QTcB), Fridericia's formula (QTcF), and/or another method, machine-read or manually over-read. The *same* QT correction formula *must* be used for *each individual participant* to determine eligibility for and discontinuation from the study. This formula may not be changed or substituted once the participant has been enrolled.
- For example, if a participant is eligible for the protocol based on QTcB, then QTcB must be used for discontinuation of this individual participant as well.
- Once the QT correction formula has been chosen for a participant's eligibility, the *same formula* must continue to be used for that participant *for all QTc data being*

*collected for data analysis*. Safety ECGs and other non-protocol specified ECGs are an exception.

- The QTc should be based on single or averaged QTc values of triplicate ECGs obtained over a brief (e.g., 5-10 minute) recording period.

A participant who meets the bulleted criteria based on the average of triplicate ECG readings will be withdrawn from study treatment:

- QTc >500 msec OR Uncorrected QT >600 msec
- Change from baseline of QTc >60 msec

Note: The baseline is defined as visit 3 pre-dose.

For participants with underlying bundle branch block, follow the discontinuation criteria listed below:

- Baseline QTc with Bundle Branch Block <450 msec, Discontinuation QTc with Bundle Branch Block >500 msec
- Baseline QTc with Bundle Branch Block <450-480 msec, Discontinuation QTc with Bundle Branch Block ≥530 msec.

See the SoA (Section 2) for data to be collected at the time of EW visit and follow-up and for any further evaluations that need to be completed.

### 8.1.3. Rechallenge

#### 8.1.3.1. Study Treatment Restart or Rechallenge

Study treatment restart or rechallenge after liver chemistry stopping criteria are met by any participant in this study is not allowed. Additionally, if hypersensitivity or anaphylactic reaction occurs, infusion should be discontinued and study restart is not allowed.

## 8.2. Withdrawal from the Study

- A participant may withdraw from the study at any time at his/her own request, or may be withdrawn at any time at the discretion of the investigator for safety, behavioral, compliance or administrative reasons.
- If the participant withdraws consent for disclosure of future information, the sponsor may retain and continue to use any data collected before such a withdrawal of consent.
- If a participant withdraws from the study, he/she may request destruction of any samples taken and not tested, and the investigator must document this in the site study records.
- Refer to the SoA (Section 2) for data to be collected at the time of early withdrawal (EW) and follow-up and for any further evaluations that need to be completed.

## 8.3. Lost to Follow Up

A participant will be considered lost to follow-up if he or she repeatedly fails to return for scheduled visits and is unable to be contacted by the study site.

The following actions must be taken if a participant fails to return to the clinic for a required study visit:

- The site must attempt to contact the participant and reschedule the missed visit as soon as possible and counsel the participant on the importance of maintaining the assigned visit schedule and ascertain whether or not the participant wishes to and/or should continue in the study.
- Before a participant is deemed lost to follow up, the investigator or designee must make every effort to regain contact with the participant (where possible, 3 telephone calls and, if necessary, a certified letter to the participant's last known mailing address or local equivalent methods). These contact attempts should be documented in the participant's medical record.
- Should the participant continue to be unreachable, he/she will be considered to have withdrawn from the study with a primary reason of lost to follow-up.

# 9. STUDY ASSESSMENTS AND PROCEDURES

- Study procedures and their timing are summarized in the SoA (Section 2).
- Protocol waivers or exemptions are not allowed
- Immediate safety concerns should be discussed with the sponsor immediately upon occurrence or awareness to determine if the participant should continue or discontinue study treatment.
- Adherence to the study design requirements, including those specified in the SoA (Section 2), is essential and required for study conduct.
- The preferred order of assessments at each visit is: ACQ-5, AQLQ, medical review and vital signs, ECG, FeNO, spirometry, blood draws and treatment administration by infusion. The preferred post-dose order of assessments is: vital signs, ECG, andblood draws.
- All screening evaluations must be completed and reviewed to confirm that potential participants meet all eligibility criteria. The investigator will maintain a screening log to record details of all participants screened and to confirm eligibility or record reasons for screening failure, as applicable.
- Procedures conducted as part of the participant's routine clinical management (eg, blood count) and obtained before signing of ICF may be utilized for screening or baseline purposes provided the procedure met the protocol-specified criteria and was performed within the time frame defined in the SoA (Section 2).

## 9.1. Efficacy Assessments

The timings of all efficacy assessments are specified in the SoA (Section 2).

### 9.1.1. Blood Eosinophils

Blood samples for haematology, specifically for blood eosinophil cell count as an efficacy endpoint, will be taken at the specified time points indicated in the SoA (Section 2). The differential cell counts will be blinded from V3 onwards.

### 9.1.2. Fractional Exhaled Nitric Oxide (FeNO)

FeNO will be measured using a handheld electronic device. Measurements will be obtained in accordance with the American Thoracic Socity (ATS)/European Respiratory Society (ERS) Recommendations for Standardized Procedures for the Online and Offline Measurement of Exhaled Lower Respiratory Nitric Oxide and Nasal Nitric Oxide American Thoracic Society; European Respiratory Society [ATS/ERS, 2005]. All sites will use standardised equipment provided by a central vendor. For each observation, at least 2 measurements will be obtained to establish reproducibility (up to 8 measurements can be performed). FeNO measurements will be interpreted in accordance with the Official ATS Clinical Practice Guideline: Interpretation of Exhaled Nitric Oxide Levels (FeNO) for Clinical Applications [Dweik, 2011].

FeNO observations must be completed before $FEV_1$ assessments.

Participants should not use their rescue medication for at least 6 hours before each FeNO assessment, unless essential for clinical need. Participants should also withhold LABAs, ICSs and OCSs for ≥1 dosing interval (i.e. ≥12 or ≥24 hours based on the prescribed dosing interval of the product) before each FeNO assessment.

Further details will be provided in the SRM and third party vendor manual.

### 9.1.3. Questionnaires

The questionnaires should be completed before any procedures are performed on the participant to avoid influencing the participant's response. To avoid biasing responses, the participant should not be told the results of diagnostic tests prior to completing the questionnaires and it is recommended that the questionnaires be administered at the same time of day during each visit (as applicable).

Adequate time must be allowed to complete all items on the questionnaires; the questionnaires must be reviewed for completeness and, if necessary, the participant must be encouraged to complete any missing assessments or items.

Instructions for completing the questionnaires can be found in the SRM.

#### 9.1.3.1. Asthma Control Questionnaire (ACQ-5)

The ACQ-5 is a five-item questionnaire that is as a measure of a participant's asthma control [Juniper, 2005]. The questions are self-completed by the participant, recalling over the previous 7 days, and enquire about the frequency and/or severity of symptoms (nocturnal awakening, activity limitation, shortness of breath and wheeze). The response options for all these questions consist of a zero ([redacted]) to six ([redacted] scale.

The questions are equally weighted and the ACQ score is the mean of the 5 questions and therefore between 0 ([redacted]) and 6 ([redacted]).

The ACQ-5 questionnaire shall be completed at the visits indicated in the SoA (Section 2).

#### 9.1.3.2. Asthma Quality of Life Questionnaire (AQLQ)

The AQLQ is a disease-specific, self-administered quality of life questionnaire recalling over the previous 14 days. It was developed to evaluate the impact of asthma treatments on the quality of life of asthma sufferers [Juniper, 1993; Juniper, 2005]. The AQLQ contains 32 items in four domains: activity limitation (11 items), symptoms (12 items), emotional function (five items), and environmental stimuli (four items). In addition, the 32 items of the questionnaire are also averaged to produce one overall quality of life score. The response format consists of a seven-point scale where a value of 1 indicates '[redacted]' and 7 indicates '[redacted]'.

The AQLQ questionnaire shall be completed at the visits indicated in the SoA (Section 2).

### 9.1.4. Spirometry

Spirometry assessments will be performed from screening through the final visit as indicated in the SoA (Section 2). The following parameters will be assessed:

- Forced expiratory volume in one second ($FEV_1$)
- Forced vital capacity (FVC)

At least three acceptable spirometry manoeuvres (from a maximum of 8 attempts) should be achieved for each spirometry assessment, in accordance with ATS/ERS standards [Miller, 2005]. At each visit, spirometry assessments must be performed at the same time of day (±1 hour) as the assessment performed at Baseline (Visit 3).

Participants should withhold use of short-acting bronchodilators for ≥6 hours and LABAs, ICSs and OCSs for ≥12 hours prior to each clinic visit, if possible. Participants who take medications that contain once daily bronchodilators and once daily maintenance therapy medication should withhold use for ≥24 hours prior to each clinic visit.

Further details will be provided in the SRM and third party vendor manual.

## 9.2. Adverse Events

The definitions of an AE or SAE can be found in Appendix 4.

The investigator and any designees are responsible for detecting, documenting, and reporting events that meet the definition of an AE or SAE and remain responsible for following up AEs that are serious, considered related to the study treatment or the study, or that caused the participant to discontinue the study treatment (see Section 8).

### 9.2.1. Time Period and Frequency for Collecting AE and SAE Information

- All SAEs will be collected from Visit 3 until the follow-up visit at the time points specified in the SoA (Section 2). At Visits 1 and 2 SAE information will be collected only for any SAEs considered as related to study participation.
- All AEs will be collected from Visit 3 until the follow-up visit at the time points specified in the SoA (Section 2).
- Medical occurrences that begin before the start of study treatment but after obtaining informed consent will be recorded on the Medical History/Current Medical Conditions section of the eCRF not the AE section.
- All SAEs will be recorded and reported to the sponsor or designee immediately and under no circumstance should this exceed 24 hours, as indicated in Appendix 4. The investigator will submit any updated SAE data to the sponsor within 24 hours of it being available.
- Investigators are not obligated to actively seek AEs or SAEs in former study participants. However, if the investigator learns of any SAE, including a death, at any time after a participant has been discharged from the study, and he/she

considers the event to be reasonably related to the study treatment or study participation, the investigator must promptly notify the sponsor.

- The method of recording, evaluating, and assessing causality of AEs and SAEs and the procedures for completing and transmitting SAE reports are provided in Appendix 4.

### 9.2.2. Method of Detecting AEs and SAEs

Care will be taken not to introduce bias when detecting AE and/or SAE. Open-ended and non-leading verbal questioning of the participant is the preferred method to inquire about AE occurrence.

### 9.2.3. Follow-up of AEs and SAEs

After the initial AE/SAE report, the investigator is required to proactively follow each participant at subsequent visits/contacts. All SAEs will be followed until the event is resolved, stabilized, otherwise explained, or the participant is lost to follow-up (as defined in Section 8.3). Further information on follow-up procedures is given in Appendix 4.

### 9.2.4. Regulatory Reporting Requirements for SAEs

- Prompt notification by the investigator to the sponsor of a SAE is essential so that legal obligations and ethical responsibilities towards the safety of participants and the safety of a study treatment under clinical investigation are met.
- The sponsor has a legal responsibility to notify both the local regulatory authority and other regulatory agencies about the safety of a study treatment under clinical investigation. The sponsor will comply with country-specific regulatory requirements relating to safety reporting to the regulatory authority, Institutional Review Boards (IRB)/Independent Ethics Committees (IEC), and investigators.
- Investigator safety reports must be prepared for suspected unexpected serious adverse reactions according to local regulatory requirements and sponsor policy and forwarded to investigators as necessary.
- An investigator who receives an investigator safety report describing a SAE or other specific safety information eg, summary or listing of SAE) from the sponsor will review and then file it along with the Investigator’s Brochure and will notify the IRB/IEC, if appropriate according to local requirements.

### 9.2.5. Cardiovascular and Death Events

For any cardiovascular events detailed in Appendix 4 and all deaths, whether or not they are considered SAEs, specific CV and Death sections of the eCRF will be required to be completed. These sections include questions regarding cardiovascular (including sudden cardiac death) and non-cardiovascular death.

The CV eCRFs are presented as queries in response to reporting of certain CV Medical Dictionary for Regulatory Activities (MedDRA) terms. The CV information should be recorded in the specific cardiovascular section of the eCRF within one week of receipt of a CV Event data query prompting its completion.

The Death eCRF is provided immediately after the occurrence or outcome of death is reported. Initial and follow-up reports regarding death must be completed within one week of when the death is reported.

### 9.2.6. Pregnancy

- Details of all pregnancies in female participants will be collected after the start of study treatment and until the follow-up visit.
- If a pregnancy is reported, the investigator should inform GSK within 2 weeks of learning of the pregnancy and should follow the procedures outlined in Appendix 5.
- Abnormal pregnancy outcomes (eg, spontaneous abortion, fetal death, stillbirth, congenital anomalies, ectopic pregnancy) are considered SAE.

## 9.3. Treatment of Overdose

An overdose is defined as a dose greater than the total doses described above which results in clinical signs and symptoms. These should be recorded by the Investigator on the AE/SAE eCRF pages. The dose of GSK3772847 considered to be an overdose has not been defined. There are no known antidotes and GSK does not recommend a specific treatment in the event of a suspected overdose. The Investigator will use clinical judgement in treating the symptoms of a suspected overdose.

In the event of an overdose, the investigator should:

1. Contact the Medical Monitor immediately.
2. Closely monitor the participant for AE/SAE and laboratory abnormalities for 16 weeks after the last dose.
3. Obtain a serum sample for PK analysis within 7 days from the date of the last dose of study treatment if requested by the Medical Monitor (determined on a case-by-case basis).
4. Document the quantity of the excess dose as well as the duration of the overdosing in the eCRF.

Decisions regarding dose interruptions or modifications will be made by the investigator in consultation with the Medical Monitor based on the clinical evaluation of the participant.

## 9.4. Safety Assessments

Planned time points for all safety assessments are provided in the SoA (Section 2).

### 9.4.1. Physical Examinations

- A complete physical examination will include, at a minimum, assessments of the Skin, Cardiovascular, Respiratory, Gastrointestinal and Neurological systems. Height and weight will also be measured and recorded.
- Investigators should pay special attention to clinical signs related to previous serious illnesses.

### 9.4.2. Vital Signs

Vital signs should be performed at the time points specified in the SoA (Section 2) prior to conducting ECG and spirometry. Blood pressure (systolic and diastolic), body temperature and pulse rate will be measured in the supine position after approximately 5 minutes rest. Body temperature will be collected in the participant's source documents only for site use. A single set of values for blood pressure and pulse rate will be collected and recorded in the source documentation and eCRF.

### 9.4.3. Electrocardiograms

All sites will use standardised ECG equipment provided by a centralised external vendor. A single 12-lead ECG and rhythm strip will be recorded after measurement of vital signs and before other clinical tests such as blood draws and pulmonary function tests. Recordings will be made at the time-points defined in the SoA (Section 2). All ECG measurements will be made with the participant in a supine position having rested in this position for approximately 5 minutes before each reading. Participants should be reminded to avoid caffeine or caffeinated drinks for at least 8 hours before each 12-lead ECG assessment. Also, decongestants are disallowed for at least 24 hours and antihistamines for at least 48 hours before each 12-lead ECG assessment.

For participants who meet the QTc, protocol defined stopping criteria, triplicate ECGs (over a brief period of time) should be performed (Section 8.1.2). The Investigator, a designated sub-Investigator or other appropriately trained site personnel will be responsible for performing each 12-lead ECG. The Investigator must provide his/her dated signature on the original paper tracing, attesting to the authenticity of the ECG machine interpretation.

All ECGs will be electronically transmitted to an independent cardiologist and evaluated. The independent cardiologist, blinded to treatment assignment, will be responsible for providing measurements of heart rate, QT intervals and an interpretation of all ECGs collected in this study. A hard copy of these results will be sent to the Investigator. The Investigator must provide his/her dated signature on the confirmed report, attesting to his/her review of the independent cardiologist's assessment.

Details of the cardiac monitoring procedures will be provided by the centralised cardiology service provider.

### 9.4.4. Continuous ambulatory ECG (Holter)

Continuous ECG monitoring (Holter) assessments have been added to the protocol to allow for a quantitative assessment of abnormal rhythm events. Holter monitors will be

provided by a third party vendor to each site. The device should be connected and electrodes attached to the participant as per the vendor's instructions.

### 9.4.5. Clinical Safety Laboratory Assessments

- Refer to Appendix 2 for the list of clinical laboratory tests to be performed and to the SoA (Section 2) for the timing and frequency.
- The investigator must review the laboratory report, document this review, and record any clinically relevant changes occurring during the study in the AE section of the CRF. The laboratory reports must be filed with the source documents. Clinically significant abnormal laboratory findings are those which are not associated with the underlying disease, unless judged by the investigator to be more severe than expected for the participant's condition.
- All laboratory tests with values considered clinically significantly abnormal during participation in the study or within 5 days after the last dose of study treatment should be repeated until the values return to normal or baseline or are no longer considered significantly abnormal by the investigator or medical monitor.
- If such values do not return to normal/baseline within a period of time judged reasonable by the investigator, the etiology should be identified and the sponsor notified.
- All protocol-required laboratory assessments, as defined in Appendix 2, must be conducted in accordance with the laboratory manual and the SoA (Section 2).
- If laboratory values from non-protocol specified laboratory assessments performed at the institution's local laboratory require a change in participant management or are considered clinically significant by the investigator (eg, SAE or AE or dose modification), then the results must be recorded in the eCRF.

### 9.4.6. Diary assessments

Participants will be issued with a diary card at visit 2. Participants should complete their daily diary pages during the study duration to record the use of rescue (SABA) medication, AEs and concomitant medication usage.

## 9.5. Pharmacokinetics

- Whole blood samples of approximately 3 mL will be collected for measurement of serum concentrations of GSK3772847as specified in the SoA (Section 2). The timing of PK samples may be altered and/or PK samples may be obtained at additional time points to ensure thorough PK monitoring. The actual date and time (24-hour clock time) of each sample will be recorded.
- Samples will be used to evaluate the PK of GSK3772847. Samples collected for analyses of serum concentration may also be used to evaluate safety or efficacy aspects related to concerns arising during or after the study.
- Instructions for the collection and handling of biological samples will be provided in the SRM.

## 9.6. Pharmacodynamics

Blood (serum) samples will be collected during this study for the purposes of measuring free and total sST2 levels. Samples will be collected at the time points indicated in the SoA (Section 2). The timing of the collections may be adjusted on the basis of emerging PK or PD data from this study or other new information in order to ensure optimal evaluation of the biomarker endpoints.

## 9.7. Genetics

Information regarding genetic/ pharmacogenetic (PGx) research is included in Appendix 6. The IEC/IRB and, where required, the applicable regulatory agency must approve the PGx and genetic assessments before these can be conducted at the site. The approval(s) must be in writing and will clearly specify approval of the PGx and genetic assessments (i.e., approval of Appendix 6).

In some cases, approval of the PGx and genetic assessments can occur after approval is obtained for the rest of the study. If so, then the written approval will clearly indicate approval of the PGx and genetic assessments is being deferred and the study, except for PGx and genetic assessments, can be initiated. When PGx and genetic assessments will not be approved, then the approval for the rest of the study will clearly indicate this and therefore, PGx and genetic assessments will not be conducted.

## 9.8. Biomarkers

### 9.8.1. Exploratory Biomarkers

Blood (serum) and induced sputum samples will be collected during this study and may be used for the purposes of measuring asthma biomarkers or endotypes of asthma, as well as response to GSK3772847. Biomarkers will include, but not be limited to, serum total IgE, fungal specific IgE (*Aspergillus fumigatus, Penicillium chrysogenum (notatum)*) as well as sputum cell counts (e.g. percentage eosinophils) and sputum levels of IL-33, IL-13, IL-4, IL-5 and TNF-α. Samples may also be used to identify factors that may influence the development of asthma and/or medically related conditions.

Samples will be collected at the time points indicated in the SoA (Section 2). Details of the sputum collection and processing methodology will be provided in the SRM.

Participants should not use their rescue medication for at least 6 hours before each sputum sampling visit, unless essential for clinical need. Participants should also withhold LABAs, ICSs and OCSs for ≥1 dosing interval (i.e. ≥12 or ≥24 hours based on the prescribed dosing interval of the product) before these visits.

### 9.8.2. Immunogenicity Assessments

Serum samples will be collected pre-dose and tested for the presence of antibodies that bind to GSK3772847, as specified in the SoA (Section 2). The actual date and time (24-hour clock time) of each sample will be recorded.

The presence of anti-GSK3772847 antibodies will be assessed using a tiered approach including a screening assay, a confirmation assay and calculation of titre.

Instructions for the collection and handling of biological samples will be provided in the SRM.

## 9.9. Medical Resource Utilization and Health Economics

Medical Resource Utilization and Health Economics parameters are not evaluated in this study.

# 10. STATISTICAL CONSIDERATIONS

The primary objective of this study is to investigate the effect of treatment with GSK3772847 compared with placebo on blood eosinophils and FeNO levels in moderate to severe asthmatic participants with AFAD currently on SoC. There are no formal hypothesis tests associated with this objective and no formal significance tests. The information acquired from this study will be used to quantify the effects that GSK3772847 has on the selected efficacy endpoints, as measured by change in the co-primary endpoints; change from baseline in blood eosinophils and change from baseline in FeNO over time.

## 10.1. Sample Size Determination

To account for a 10% withdrawal rate, approximately 46 participants will be randomised in 1:1 ratio to active arm or placebo arm, to ensure approximately 40 will be evaluable in total.

The sample size is based on feasibility and no formal sample size calculation has been performed.

As described below, change from baseline in blood eosinophils and change from baseline in FeNO will be log-transformed.

Estimates of the variability for the ratio to baseline in blood eosinophils and the ratio to baseline in FeNO were obtained from [Anderson, 2012] and from studies in Mepolizumab in participants with severe asthma.

The half widths of the 95% confidence interval of the point estimate of the treatment ratio of active to placebo for the ratio to baseline in blood eosinophils and FeNO have been calculated and are displayed in Table 4 and Table 5.

**Table 4 Expected Half Width of 95% Confidence Interval for the ratio of active to placebo of ratio to baseline in blood eosinophils with an estimated between participant standard deviation (log-scale) of 0.9.**

| Sample size (per arm) | Expected Half Width of 95% Confidence Interval for the ratio of active to placebo |
|---|---|
| 15 | 96% |
| 20 | 78% |
| 25 | 67% |
| 30 | 59% |

Assuming a standard deviation of 0.9 (log-scale), and a sample size of 20 participants per treatment arm, it is estimated that the lower and upper bounds of the 95% confidence interval for the ratio of active to placebo of ratio to baseline in blood eosinophils will be within approximately 78% of the ratio i.e. if the observed ratio was 1.5 then the confidence interval would be approximately 0.84 to 2.67.

**Table 5 Expected Half Width of 95% Confidence Interval for the ratio of active to placebo of ratio to baseline in fractional exhaled nitric oxide with an estimated between participant standard deviation (log-scale) of 0.43.**

| Sample size (per arm) | Expected Half Width of 95% Confidence Interval for the ratio of active to placebo |
|---|---|
| 15 | 38% |
| 20 | 32% |
| 25 | 28% |
| 30 | 25% |

For ratio to baseline in FeNO over time, assuming a standard deviation (log-scale) of 0.43, and a sample size of 20 participants per treatment arm it is estimated that the lower and upper bounds of the 95% confidence interval for the ratio of active to placebo will be within approximately 32% of the ratio. i.e. if the observed ratio was 1.5 the confidence interval would be approximately 1.14 to 1.98

A sample size of 20 participants per treatment arm will give us an adequate level of precision of the treatment effect differences.

To evaluate study success criteria, data from a bivariate normal distribution will be simulated under different treatment effect assumptions for different sample sizes. The samples from the posterior probability distribution from the MCMC approximation will be divided into different regions exploring different treatment cut-off values. Further, the multivariate case will also be explored involving AQLQ or ACQ-5. The prior for the treatment difference for FeNO and eosinophils was elicited at a prior elicitation exercise with internal experts at GSK. This prior for the treatment difference includes the experts' uncertainty about the size of the mean treatment effect.

## 10.2. Populations for Analyses

For purposes of analysis, the following populations are defined:

| Population | Description |
|---|---|
| Enrolled | All participants who sign the ICF |
| Randomised | All participants who were randomised. A participant who is recorded as a screen or run-in failure and also randomised will be considered to be randomised in error provided they have not performed any study assessments. |
| Modified Intent-to-treat | All randomised participants who take at least 1 dose of study treatment.<br>Participants will be analysed according to the treatment they actually received |
| Safety | This population will be the same as the Modified Intent-to-treat population. |
| PK | All randomised participants who received at least one dose of study medication, and for whom at least one pharmacokinetic sample was obtained, analysed and was measurable. |

## 10.3. Statistical Analyses

### 10.3.1. Efficacy Analyses

All efficacy analyses will be performed on the Intent-to-Treat population.

| Endpoint | Statistical Analysis Methods |
|---|---|
| Primary | The co-primary endpoints are blood eosinophils and FeNO. Blood eosinophils and FeNO will be log-transformed prior to analysis. Change from baseline in log transformed blood eosinophils will be analysed using a mixed model repeated measures analysis (MMRM), where baseline is defined as Week 0. The model will include fixed effects of treatment group, which strata the participant was in (i.e. whether the participant was taking antifungals or not), log-transformed baseline blood eosinophils, time as a categorical variable and a treatment by time interaction. Participant will be fitted as a random effect, and time as a repeated effect. A similar analysis will be done for change from baseline in log-transformed FeNO, except log-transformed baseline FeNO will be used (rather than log-transformed baseline blood eosinophils).<br>The estimated treatment ratios together with 95% CIs (back-transformed from the differences on the log-scale) will be presented at the end of weeks 2, 4, 8, and 12 for each of FeNO and blood eosinophils.<br><br>To calculate the probability of success, accounting for the correlation between endpoints, the joint posterior probability that the true ratios for both endpoints simultaneously lie to the left hand side of unity will be estimated. This will be obtained at week 12. |
| Secondary | Change from baseline in ACQ-5 absolute score and AQLQ at Weeks 2, 4, 8 and 12 will be separately analysed using a MMRM for each endpoint, where baseline is defined as Week 0. The model for ACQ-5 will include fixed effects of treatment group, which strata the participant was in (i.e. whether the participant was taking antifungals or not),baseline ACQ-5, time as a categorical variable and a treatment by time interaction. Participant will be fitted as a random effect and time as a repeated effect. A similar analysis will be done for Change from baseline in AQLQ total and domain scores at Weeks 4, 8 and 12, except baseline AQLQ (total or domain score) will be used instead of ACQ-5. The estimated treatment differences together with the 95% CIs will be presented at the end of weeks 4, 8 and 12 for each of ACQ-5 and AQLQ.<br><br>Change from baseline in pre-bronchodilator FEV1 will be listed and summarised.<br><br>Analyses of responders to ACQ-5 and AQLQ will be described in the Reporting Analysis Plan (RAP).<br><br>Further analysis on the secondary endpoints will be described in the RAP. |
| Exploratory | Will be described in the reporting and analysis plan |

### 10.3.2. Safety Analyses

All safety analyses will be performed on the Safety Population.

| Endpoint | Statistical Analysis Methods |
|---|---|
| Primary | There is no primary safety analysis. |
| Secondary | The following secondary safety endpoints will be analysed descriptively by treatment group:<br>• Treatment emergent AE<br>• Clinical Laboratory safety data<br>• Vital signs (blood pressure, heart rate)<br>• 12–Lead ECG monitoring<br>• 24-hour Holter monitoring<br>• Incidence and titres of serum anti-GSK3772847 antibodies post dosing<br>Details will be described in the reporting and analysis plan |
| Exploratory | Will be described in the reporting and analysis plan |

Adverse events (AEs) will be coded using the standard GSK dictionary, MedDRA, and grouped by body system. The number and percentage of participants experiencing at least one AE of any type, AEs within each body system and AEs within each preferred term will be presented for each treatment group. Separate summaries will be provided for all AEs, drug related AEs, fatal AEs, non-fatal SAEs, adverse events of special interest and AEs leading to withdrawal. Deaths and SAEs, if applicable, will be documented in case narrative format.

### 10.3.3. Pharmacokinetic Analyses

All pharmacokinetic analyses will be performed on the Pharmacokinetic Population.

| Endpoint | Statistical Analysis Methods |
|---|---|
| Primary | There is no primary pharmacokinetic analysis. |
| Secondary | The serum GSK3772847 levels from this study will be summarised by treatment and nominal time.<br>Further details will be described in the report and analysis plan. |

### 10.3.4. Pharmacodynamic Analyses

All pharmacodynamic analyses will be described in reporting and analysis plan.

### 10.3.5. Other Analyses

PK, PD and biomarker exploratory analyses will be described in the reporting and analysis plan. Any population PK/PKPD analyses will be presented separately from the main clinical study report (CSR).

### 10.3.6. Interim Analyses

An interim analysis may be conducted for internal decision making. Access to the unblinded interim data and the interim results will be restricted. No changes to the study design will be made based on the interim analysis results. Full details of the interim analyses including details of who will have access to the interim data and interim results will be provided in an interim analysis plan.

**Instream review:**
An iSRC (see Appendix 3) will periodically review unblinded safety data to protect and maintain participant safety whilst maintaining scientific validity. Members of the iSRC will be independent of the project. The data will include, but not necessarily be limited to SAEs, Holters and ECGs. Details are described in the Charter.

# 11. REFERENCES

Aburuz S, Gamble J, Heaney CG. Assessment of impairment in heatlh-related quality of life in patients with difficult asthma: Psychometric performance of the Asthma Quality of Life Questionnaire. *Respirology* 2007; 12:227-233.

American Thoracic Society; European Respiratory Society. ATS/ERS recommendations for standardized procedures for the online and offline measurement of exhaled lower respiratory nitric oxide and nasal nitric oxide. *Am J Respir Crit Care Med* 2005; 171: 912-930.

Anderson WJ, Short PM, Williamson PA, Lipworth BJ. Inhaled Corticosteroid Dose Response Using Domiciliary Exhaled Nitric Oxide The FeNo Type Trial. *Chest* 2012; 142: 1553–1561.

Arshad M I, Khan HA, Noel G , Piquet-Pellorce C, Samson M. Potential therapeutic effects of alarmin cytokine interleukin 33 or its inhibitors in various diseases. *Clin Therapeutics* 2016; 38:100-116.

Castanhinha S, Sherburn R, Walker S, Gupta A, Bossley CJ, Buckley J, et al. Pediatric severe asthma with fungal sensitization is mediated by steroid-resistant IL-33. *J Allergy Clin Immunol* 2015; 136: 312-322.

Chung, KF, Wenzel SE, Brozek JL, Bush A, Castro M, Sterk PJ, et al. International ERS/ATS guidelines on definition, evaluation and treatment of severe asthma. *Eur Respir J* 2014; 43:343-373.

Denning DW, O'Driscoll BR, Hogaboam CM, Bowyer P, Niven R. The link between fungi and severe asthma: a summary of the evidence. *Eur Respir J* 2006; 27:615-626.

Dweik RA, Boggs PB, Erzurum SC, Irvin CG, Leigh MW, Lundberg JO, et al. An official ATS clinical practice guideline: interpretation of exhaled nitric oxide levels (FENO) for clinical applications. *Am J Respir Crit Care Med* 2011; 184:602-615.

GlaxoSmithKline Document Number: 2017N316832_00;Investigator's Brochure for GSK3772847 07 June 2017

GlaxoSmithKline Document Number: 2017N344518_00, Clinical Study Report for GSK3772847, ; 20 Sep 2017

Global Initiative for Asthma, GINA. 2017. *GLOBAL STRATEGY FOR ASTHMA MANAGEMENT AND PREVENTION.* Available from: www.ginasthma.org.

Juniper EF, Guyatt GH, Williams A, Griffith LE. Measuring quality of life in asthma. *Am Rev Respir Dis* 1993; 147:832-838.

Juniper EF, Svensson K, Mork AC, Stahl E. Measurement properties and interpretation of three shortened versions of the asthma control questionaire. *Respir Med* 2005; 99:553-558.

Juniper EF, Svensson K, Mork AC, Stahl E. Modification of the Asthma Quality of Life Questionnaire (standardised) in patients 12 years and older. *Health and Quality of Life Outcomes* 2005; 3:58.

Menzies D, Holmes L, McCumesky G, Prys-Picard C, Niven R. Aspergillus sensitization is associated with airflow limitation and bronchiectasis in severe asthma. *Allergy* 2011; 66:679-685.

Miller MR, Hankinson J, Brusasco V, Burgos F, Casaburi R, Coates A, et al.. Standardisation of spirometry. *Eur Respir J* 2005; 26:319-398.

Moore WC, Bleecker ER, Curran-Everett D, Erzurum SC, Ameredes BT, Bacharier L, et al. Characterization of the severe asthma phenotype by the National Heart, Lung, and Blood Institute's Severe Asthma Research Program. *J Allergy Clin Immunol* 2007; 119:405-413.

Moss RB. Treatment options in severe fungal asthma and allergic bronchopulmonary aspergillosis. *European Respiratory Journal* 2014; 1487–1500.

O'Driscoll BR, Hopkinson LC, Denning DW. Mold sensitisation is common amongst patients with severe asthma requiring multiple hospital admissions. *BioMed Central Pulmonary Medicine* 2005; 5:4.

Oshikawa K, Kuroiwa K, Tago K, Iwahana H, Yanagisawa K, Ohno S, et al. Elevated soluble ST2 protein levels in sera of patients with asthma with an acute exacerbation. *Am J Respir Crit Care Med* 2001; 164:277-281.

Quanjer PH, Stanojevic S, Cole TJ, Baur X, Hall GL, Culver BH, et al. Multi-Ethnic Reference Values For Spirometry For The 3–95 Year Age Range: The Global Lung Function 2012 Equations. *Eur Respir J.* 2012; 40:1324–1343.

Rick EM, Woolnough K, Pashley CH, Wardlaw AJ. Allergic Fungal Airway Disease. *J Investig Allergol Clin Immunol* 2016; 26:344-354.

Smithgall MD, Comeau MR, Yoon BR, Kaufman D, Armitage R, Smith DE. IL-33 amplifies both Th1- and Th2-type responses through its activity on human basophils, allergen-reactive Th2 cells, iNKT and NK cells. *International Immunology* 2008; 20:1019-1030.

Tracy M, Okorie C, Foley E, Moss R. Allergic Bronchopulmonary Aspergillosis. *Journal of Fungi* 2016; 17.

Woolnough KF, Richardson M, Newby C, Craner M, Bourne M, Monteiro W, et al. The relationship between biomarkers of fungal allergy and lung damage in asthma. *Clinical & Experimental Allergy* 2016; 47:48-56.

Yagami A, Orihara K, Morita H, Futamura K, Hashimoto N, Matsumoto K, et al. IL-33 mediates inflammatory responses in human lung tissue cells. *J Immunol* 2010; 185:5743-5750.

# 12. APPENDICES

## 12.1. Appendix 1: Abbreviations and Trademarks

### Abbreviations

| | |
|---|---|
| ABPA | Allergic bronchopulmonary aspergillosis |
| ACQ | Asthma Control Questionnaire |
| AE | Adverse Event |
| AFAD | Allergic fungal airway disease |
| ALT | Alanine Transaminase |
| AQLQ | Asthma Quality of Life Questionnaire |
| AST | Aspartate Transaminase |
| ATS | American Thoracic Society |
| AUC | Area under the curve |
| AV | Atrioventricular |
| BAL | Bronchoalveolar lavage |
| bpm | Beats per minute |
| CAD | Coronary artery disease |
| CPK | Creatine phosphokinase |
| CRS | Cytokine release syndrome |
| CSR | Clinical study report |
| CV | Cardiovascular |
| ECG | Electrocardiogram |
| (e)CRF | (Electronic) Case Report Form |
| ERS | European Respiratory Society |
| EU | European Union |
| EW | Early Withdrawal |
| FeNO | Fractional exhaled Nitric Oxide |
| $FEV_1$ | Forced expiratory volume in 1 second |
| FVC | Forced vital capacity |
| GCP | Good clinical practice |
| GINA | Global Initiative for Asthma |
| GLP | Good laboratory practice |
| GSK | GlaxoSmithKline |
| HBsAg | Hepatitis B surface antigen |
| HIV | Human immunodeficiency virus |
| HR | Heart rate |
| HRCT | High-resolution computed tomography |
| HRT | Hormone replacement therapy |
| IB | Investigator's Brochure |
| ICF | Informed consent form |
| ICH | International Conference on Harmonization |

| | |
|---|---|
| ICS | Inhaled corticosteroids |
| IEC | Independent Ethics Committee |
| IgE | Immunoglobulin E |
| IgG2σ | human immunoglobulin G2 sigma isotype |
| IL-1RL1 | Interleukin-1 receptor like-1 |
| IL-33R | Interleukin-33 receptor |
| INR | International normalized ratio |
| IP | Investigational Product |
| IRB | Institutional Review Board |
| iSRC | Internal safety review committee |
| IV | Intravenous |
| IWRS | Interactive web response system |
| LABA | Long-acting beta-2-agonist |
| LTRA | Leukotriene receptor antagonist |
| MedDRA | Medical Dictionary for Regulatory Activities |
| MM | Michaelis Menten |
| MMRM | Mixed model repeated measures analysis |
| MMRM | Mixed model repeated measures analysis |
| NT-proBNP | N-terminal prohormone of brain natriuretic peptide |
| NYHA | New York Heart Association |
| PD | Pharmacodynamic |
| PGx | Pharmacogenetic |
| PK | Pharmacokinetic |
| PSVT | Paroxysmal supraventricular tachycardia |
| QoL | Quality of Life |
| QTc | QT interval corrected for heart rate |
| QTcB | QT interval corrected for heart rate by Bazett's formula |
| QTcF | QT interval corrected for heart rate by Fridericia's formula |
| RAP | Reporting Analysis Plan |
| SABA | Short acting β-2 agonist |
| SAE | Serious adverse event |
| SC | Subcutaneous |
| SoA | Schedule of activities |
| SoC | Standard of care |
| SRM | Study Reference Manual |
| (s) ST2 | (soluble) Suppressor of tumorigenicity 2 |
| T2 | Type 2 |
| ULN | Upper limit of normal |
| VT | Ventricular tachycardia |
| WOCBP | woman of childbearing potential |

## Trademark Information

| Trademarks of the GlaxoSmithKline group of companies |
| --- |
| NONE |

| Trademarks not owned by the GlaxoSmithKline group of companies |
| --- |
| None |

## 12.2. Appendix 2: Clinical Laboratory Tests

- The tests detailed in Table 6 will be performed by the central laboratory.
- All protocol required laboratory assessments (haematology, clinical chemistry and urinalysis) must be conducted in accordance with the Laboratory Manual and the SoA (Section 2). Laboratory requisition forms must be completed and samples must be clearly labelled with the participant number, protocol number, site/centre number, and visit date. Details for the preparation and shipment of samples will be provided by the laboratory and are detailed in the Laboratory Manual. Reference for all safety parameters will be provided to the site by the laboratory responsible for the assessments.
- All blood samples which will be taken pre-dose, will be sent to a central laboratory for analysis (details provided in the Laboratory Manual). Standard reference ranges will be used.
- If additional non-protocol specified laboratory assessments are performed at the institution's local laboratory and result in a change in participant management or are considered clinically significant by the Investigator (e.g., SAE or AE or dose modification), the results must be recorded in the eCRF.
- Refer to the Laboratory Manual for appropriate processing and handling of samples to avoid duplicate and/or additional blood draws.
- Protocol-specific requirements for inclusion or exclusion of participants are detailed in Section 6 of the protocol.
- Additional tests may be performed at any time during the study as determined necessary by the investigator or required by local regulations.

**Table 6 Protocol-Required Safety Laboratory Assessments**

| Laboratory Assessments | Parameters | | | |
|---|---|---|---|---|
| Haematology | Platelet Count<br>RBC Count<br>Haemoglobin<br>Haematocrit<br>White Cell Count<br>RDW | RBC Indices:<br>MCV<br>MCH<br>MCHC | WBC count with Differential:<br>Neutrophils<br>Lymphocytes<br>Monocytes<br>Eosinophils<br>Basophils<br>These will be blinded from V3 onwards. | |
| Clinical Chemistry[1] | BUN | Potassium | Aspartate Aminotransferase (AST)/ Serum Glutamic-Oxaloacetic Transaminase | Total and direct bilirubin |
| | Creatinine | Sodium | ALT/ Serum Glutamic-Pyruvic Transaminase (SGPT) | Total Protein |
| | Glucose nonfasting | Calcium | Alkaline phosphatase | Creatine phosphokinase (CPK) |
| | Albumin | Phosphorus | GGT | Chloride |
| | | Carbon Dioxide | | |
| Serum IgE | • Total IgE<br>• Specific IgE to *Aspergillus fumigatus and Penicillium chrysogenum (notatum)*<br>These will be blinded from V3 onwards. | | | |
| Cardiac Markers | • Cardiac troponin I (cTN I)<br>• NT-proBNP | | | |
| Routine Urinalysis | • Specific gravity<br>• pH, glucose, protein, blood, ketones, bilirubin, leukocyte, nitrite, urobilinogen by dipstick<br>• Microscopic examination (if blood or protein is abnormal) | | | |
| Other Screening Tests | • Follicle-stimulating hormone and estradiol (as needed in women of non-childbearing potential only) | | | |

| Laboratory Assessments | Parameters |
|---|---|
| | • Serum or urine human chorionic gonadotropin (hCG) pregnancy test (as needed for women of childbearing potential)[2]<br>• Serology [(HIV antibody, HBsAg, and hepatitis C virus antibody)<br>All study-required laboratory assessments will be performed by a central laboratory. |

NOTES :

1. Details of liver chemistry stopping criteria and required actions and follow-up assessments after liver stopping or monitoring event are given in Section 8.1 and Appendix 7 All events of ALT ≥3 × upper limit of normal (ULN) and bilirubin ≥2 × ULN (>35% direct bilirubin) or ALT ≥3 × ULN and international normalized ratio (INR) >1.5, if INR measured, which may indicate severe liver injury (possible Hy's Law), must be reported as an SAE (excluding studies of hepatic impairment or cirrhosis).
2. Local urine testing will be standard for the protocol unless serum testing is required by local regulation or IRB/IEC.

Abbreviations: RBC= Red Blood Cell Count, WBC= White Blood Cell Count, MCV= Mean corpuscular volume, MCH= mean corpuscular haemoglobin, MCHC= mean corpuscular haemoglobin concentration, RDW= Red cell distribution width, AST= Aspartate Aminotransferase, ALT= Alanine Aminotransferase, SGPT= Serum Glutamic- Oxaloacetic Transaminase, CPK= creatine phosphokinase, GGT= Gamma-glutamyltransferase, hCG= human chorionic gonadotropin, HIV= Human Immunodeficiency Virus

Blood eosinophils, total IgE and specific IgE from V3 only that could unblind the study will not be reported to investigative sites or other blinded personnel until the study has been unblinded.

## 12.3. Appendix 3: Study Governance Considerations

### Regulatory and Ethical Considerations

- This study will be conducted in accordance with the protocol and with:
    - Consensus ethical principles derived from international guidelines including the Declaration of Helsinki and Council for International Organizations of Medical Sciences International Ethical Guidelines
    - Applicable International Conference on Harmonization (ICH) Good Clinical Practice (GCP) Guidelines
    - Applicable laws and regulations
- The protocol, protocol amendments, ICF, Investigator Brochure, and other relevant documents (eg, advertisements) must be submitted to an IRB/IEC by the investigator and reviewed and approved by the IRB/IEC before the study is initiated.
- Any amendments to the protocol will require IEC/IRB approval before implementation of changes made to the study design, except for changes necessary to eliminate an immediate hazard to study participants.
- The investigator will be responsible for the following:
    - Providing written summaries of the status of the study to the IRB/IEC annually or more frequently in accordance with the requirements, policies, and procedures established by the IRB/EC
    - Notifying the IRB/IEC of SAE or other significant safety findings as required by IRB/IEC procedures
    - Providing oversight of the conduct of the study at the site and adherence to requirements of 21 CFR, ICH guidelines, the IRB/IEC, European regulation 536/2014 for clinical studies (if applicable), and all other applicable local regulations

### Financial Disclosure

Investigators and sub-investigators will provide the sponsor with sufficient, accurate financial information as requested to allow the sponsor to submit complete and accurate financial certification or disclosure statements to the appropriate regulatory authorities. Investigators are responsible for providing information on financial interests during the course of the study and for 1 year after completion of the study.

### Informed Consent Process

- The investigator or his/her representative will explain the nature of the study to the participant or his/her legally authorised representative and answer all questions regarding the study.
- Participants must be informed that their participation is voluntary. Participants or their legally authorised representative will be required to sign a statement of

informed consent that meets the requirements of 21 CFR 50, local regulations, ICH guidelines, Health Insurance Portability and Accountability Act requirements, where applicable, and the IRB/IEC or study centre.

- The medical record must include a statement that written informed consent was obtained before the participant was enrolled in the study and the date the written consent was obtained. The authorised person obtaining the informed consent must also sign the ICF.
- Participants must be re-consented to the most current version of the ICF(s) during their participation in the study.
- A copy of the ICF(s) must be provided to the participant or the participant's legally authorised representative.
- Participants who are rescreened are required to sign a new ICF.

## Data Protection

- Participants will be assigned a unique identifier by the sponsor. Any participant records or datasets that are transferred to the sponsor will contain the identifier only; participant names or any information which would make the participant identifiable will not be transferred.
- The participant must be informed that his/her personal study-related data will be used by the sponsor in accordance with local data protection law. The level of disclosure must also be explained to the participant.
- The participant must be informed that his/her medical records may be examined by Clinical Quality Assurance auditors or other authorised personnel appointed by the sponsor, by appropriate IRB/IEC members, and by inspectors from regulatory authorities.

## Committees Structure

The Internal Safety Review Committee (iSRC) consists of a panel of four members: two senior GSK members and two medical consultants. The members are all independent from this study and their remit is to periodically review specific aspects of the clinical trial in order to protect and enhance patient safety while maintaining the scientific validity of the data.

The iSRC will meet at predefined times as defined in the iSRC charter to evaluate cardiac safety data for participants who receive GSK3774827.

## Publication Policy

- The results of this study may be published or presented at scientific meetings. If this is foreseen, the investigator agrees to submit all manuscripts or abstracts to the sponsor before submission. This allows the sponsor to protect proprietary information and to provide comments.
- The sponsor will comply with the requirements for publication of study results. In accordance with standard editorial and ethical practice, the sponsor will

generally support publication of multicentre studies only in their entirety and not as individual site data. In this case, a coordinating investigator will be designated by mutual agreement.

- Authorship will be determined by mutual agreement and in line with International Committee of Medical Journal Editors authorship requirements.

### Dissemination of Clinical Study Data

- Where required by applicable regulatory requirements, an investigator signatory will be identified for the approval of the clinical study report. The investigator will be provided reasonable access to statistical tables, figures, and relevant reports and will have the opportunity to review the complete study results at a GSK site or other mutually-agreeable location.
- GSK will also provide the investigator with the full summary of the study results. The investigator is encouraged to share the summary results with the study subjects, as appropriate.
- GSK will provide the investigator with the randomisation codes for their site only after completion of the full statistical analysis.

### Data Quality Assurance

- All participant data relating to the study will be recorded on printed or electronic CRF unless transmitted to the sponsor or designee electronically (eg, laboratory data). The investigator is responsible for verifying that data entries are accurate and correct by physically or electronically signing the eCRF.
- The investigator must maintain accurate documentation (source data) that supports the information entered in the eCRF.
- The investigator must permit study-related monitoring, audits, IRB/IEC review, and regulatory agency inspections and provide direct access to source data documents.
- The sponsor or designee is responsible for the data management of this study including quality checking of the data.
- Study monitors will perform ongoing source data verification to confirm that data entered into the eCRF by authorized site personnel are accurate, complete, and verifiable from source documents; that the safety and rights of participants are being protected; and that the study is being conducted in accordance with the currently approved protocol and any other study agreements, ICH GCP, and all applicable regulatory requirements.
- Records and documents, including signed ICF, pertaining to the conduct of this study must be retained by the investigator for 25 years from the issue of the final CSR/ equivalent summary unless local regulations or institutional policies require a longer retention period. No records may be destroyed during the retention period without the written approval of the sponsor. No records may be transferred to another location or party without written notification to the sponsor.

## Source Documents

- Source documents provide evidence for the existence of the participant and substantiate the integrity of the data collected. Source documents are filed at the investigator's site.
- Data reported on the CRF or entered in the eCRF that are transcribed from source documents must be consistent with the source documents or the discrepancies must be explained. The investigator may need to request previous medical records or transfer records, depending on the study. Also, current medical records must be available.
- Definition of what constitutes source data can be found in the source document agreement (to be signed by the investigator (or delegate) at each site) .

## Study and Site Closure

GSK or its designee reserves the right to close the study site or terminate the study at any time for any reason at the sole discretion of GSK. Study sites will be closed upon study completion. A study site is considered closed when all required documents and study supplies have been collected and a study-site closure visit has been performed.

The investigator may initiate study-site closure at any time, provided there is reasonable cause and sufficient notice is given in advance of the intended termination.

Reasons for the early closure of a study site by the sponsor or investigator may include but are not limited to:

- Failure of the investigator to comply with the protocol, the requirements of the IRB/IEC or local health authorities, the sponsor's procedures, or GCP guidelines
- Inadequate recruitment of participants by the investigator
- Discontinuation of further study treatment development

## 12.4. Appendix 4: Adverse Events: Definitions and Procedures for Recording, Evaluating, Follow-up, and Reporting

### Definition of AE

| AE Definition |
| --- |
| • An AE is any untoward medical occurrence in a clinical study participant, temporally associated with the use of a study treatment, whether or not considered related to the study treatment.<br>• NOTE: An AE can therefore be any unfavorable and unintended sign (including an abnormal laboratory finding), symptom, or disease (new or exacerbated) temporally associated with the use of a study treatment. |

| Events Meeting the AE Definition |
| --- |
| • Any abnormal laboratory test results (haematology, clinical chemistry, or urinalysis) or other safety assessments (eg, ECG, radiological scans, vital signs measurements), including those that worsen from baseline, considered clinically significant in the medical and scientific judgment of the investigator (ie, not related to progression of underlying disease).<br>• Exacerbation of a chronic or intermittent pre-existing condition including either an increase in frequency and/or intensity of the condition.<br>• New conditions detected or diagnosed after study treatment administration even though it may have been present before the start of the study.<br>• Signs, symptoms, or the clinical sequelae of a suspected drug-drug interaction.<br>• Signs, symptoms, or the clinical sequelae of a suspected overdose of either study treatment or a concomitant medication. Overdose per se will not be reported as an AE/SAE unless it is an intentional overdose taken with possible suicidal/self-harming intent. Such overdoses should be reported regardless of sequelae.<br>• "Lack of efficacy" or "failure of expected pharmacological action" per se will not be reported as an AE or SAE. Such instances will be captured in the efficacy assessments. However, the signs, symptoms, and/or clinical sequelae resulting from lack of efficacy will be reported as AE or SAE if they fulfil the definition of an AE or SAE. |

| Events NOT Meeting the AE Definition |
| --- |
| • Any clinically significant abnormal laboratory findings or other abnormal safety assessments which are associated with the underlying disease, unless judged by the investigator to be more severe than expected for the participant's condition.<br>• The disease/disorder being studied or expected progression, signs, or symptoms of |

| |
|---|
| the disease/disorder being studied, unless more severe than expected for the participant's condition.<br>• Medical or surgical procedure (eg, endoscopy, appendectomy): the condition that leads to the procedure is the AE.<br>• Situations in which an untoward medical occurrence did not occur (social and/or convenience admission to a hospital).<br>• Anticipated day-to-day fluctuations of pre-existing disease(s) or condition(s) present or detected at the start of the study that do not worsen. |

## Definition of SAE

If an event is not an AE per definition above, then it cannot be an SAE even if serious conditions are met (e.g., hospitalisation for signs/symptoms of the disease under study, death due to progression of disease).

| **A SAE is defined as any untoward medical occurrence that, at any dose:** |
|---|
| **a. Results in death** |
| **b. Is life-threatening**<br>The term 'life-threatening' in the definition of 'serious' refers to an event in which the participant was at risk of death at the time of the event. It does not refer to an event, which hypothetically might have caused death, if it were more severe. |
| **c. Requires inpatient hospitalisation or prolongation of existing hospitalisation**<br>In general, hospitalisation signifies that the participant has been detained (usually involving at least an overnight stay) at the hospital or emergency ward for observation and/or treatment that would not have been appropriate in the physician's office or outpatient setting. Complications that occur during hospitalisation are AE. If a complication prolongs hospitalisation or fulfils any other serious criteria, the event is serious. When in doubt as to whether "hospitalisation" occurred or was necessary, the AE should be considered serious.<br><br>Hospitalisation for elective treatment of a pre-existing condition that did not worsen from baseline is not considered an AE. |
| **d. Results in persistent disability/incapacity**<br>• The term disability means a substantial disruption of a person's ability to conduct normal life functions.<br>• This definition is not intended to include experiences of relatively minor medical significance such as uncomplicated headache, nausea, vomiting, diarrhoea, influenza, and accidental trauma (e.g., sprained ankle) which may interfere with or prevent everyday life functions but do not constitute a substantial disruption. |
| **e. Is a congenital anomaly/birth defect** |

| | |
|---|---|
| **f.** | **Other situations:** |
| • | Medical or scientific judgment should be exercised in deciding whether SAE reporting is appropriate in other situations such as important medical events that may not be immediately life-threatening or result in death or hospitalisation but may jeopardise the participant or may require medical or surgical intervention to prevent one of the other outcomes listed in the above definition. These events should usually be considered serious.<br>Examples of such events include invasive or malignant cancers, intensive treatment in an emergency room or at home for allergic bronchospasm, blood dyscrasias or convulsions that do not result in hospitalisation, or development of drug dependency or drug abuse. |

## Definition of Cardiovascular Events

| **Cardiovascular Events (CV) Definition:** |
|---|
| Investigators will be required to fill out the specific CV event page of the eCRF for the following AEs and SAEs:<br>• Myocardial infarction/unstable angina<br>• Congestive heart failure<br>• Arrhythmias<br>• Valvulopathy<br>• Pulmonary hypertension<br>• Cerebrovascular events/stroke and transient ischemic attack<br>• Peripheral arterial thromboembolism<br>• Deep venous thrombosis/pulmonary embolism<br>• Revascularization |

## Recording AE and SAE

| **AE and SAE Recording** |
|---|
| • When an AE/SAE occurs, it is the responsibility of the investigator to review all documentation (eg, hospital progress notes, laboratory, and diagnostics reports) related to the event.<br>• The investigator will then record all relevant AE/SAE information in the eCRF.<br>• It is **not** acceptable for the investigator to send photocopies of the participant’s medical records to GSK in lieu of completion of the GSK /AE/SAE eCRF page.<br>• There may be instances when copies of medical records for certain cases are |

requested by GSK. In this case, all participant identifiers, with the exception of the participant number, will be redacted on the copies of the medical records before submission to GSK.

- The investigator will attempt to establish a diagnosis of the event based on signs, symptoms, and/or other clinical information. Whenever possible, the diagnosis (not the individual signs/symptoms) will be documented as the AE/SAE.

**Assessment of Intensity**

The investigator will make an assessment of intensity for each AE and SAE reported during the study and assign it to 1 of the following categories:

- Mild: An event that is easily tolerated by the participant, causing minimal discomfort and not interfering with everyday activities.
- Moderate: An event that causes sufficient discomfort and interferes with normal everyday activities.
- Severe: An event that prevents normal everyday activities. An AE that is assessed as severe should not be confused with an SAE. Severe is a category utilised for rating the intensity of an event; and both AE and SAE can be assessed as severe.

  An event is defined as 'serious' when it meets at least 1 of the predefined outcomes as described in the definition of an SAE, NOT when it is rated as severe.

**Assessment of Causality**

- The investigator is obligated to assess the relationship between study treatment and each occurrence of each AE/SAE.
- A "reasonable possibility" of a relationship conveys that there are facts, evidence, and/or arguments to suggest a causal relationship, rather than a relationship cannot be ruled out.
- The investigator will use clinical judgment to determine the relationship.
- Alternative causes, such as underlying disease(s), concomitant therapy, and other risk factors, as well as the temporal relationship of the event to study treatment administration will be considered and investigated.
- The investigator will also consult the IB and/or Product Information, for marketed products, in his/her assessment.
- For each AE/SAE, the investigator **must** document in the medical notes that he/she has reviewed the AE/SAE and has provided an assessment of causality.
- There may be situations in which an SAE has occurred and the investigator has minimal information to include in the initial report to GSK. However, **it is very important that the investigator always make an assessment of causality for every event before the initial transmission of the SAE data to** GSK.

| |
|---|
| • The investigator may change his/her opinion of causality in light of follow-up information and send an SAE follow-up report with the updated causality assessment.<br>• The causality assessment is one of the criteria used when determining regulatory reporting requirements. |

| Follow-up of AE and SAE |
|---|
| • The investigator is obligated to perform or arrange for the conduct of supplemental measurements and/or evaluations as medically indicated or as requested by GSK to elucidate the nature and/or causality of the AE or SAE as fully as possible. This may include additional laboratory tests or investigations, histopathological examinations, or consultation with other health care professionals.<br>• If a participant dies during participation in the study or during a recognised follow-up period, the investigator will provide GSK with a copy of any post-mortem findings including histopathology.<br>• New or updated information will be recorded in the originally completed eCRF.<br>• The investigator will submit any updated SAE data to GSK within 24 hours of receipt of the information. |

### Reporting of SAE to GSK

| SAE Reporting to GSK via Electronic Data Collection Tool |
|---|
| • The primary mechanism for reporting SAE to GSK will be the electronic data collection tool.<br>• If the electronic system is unavailable for more than 24 hours, then the site will use the paper SAE data collection tool (see next section).<br>• The site will enter the SAE data into the electronic system as soon as it becomes available.<br>• The investigator or medically-qualified sub-investigator must show evidence within the eCRF (e.g., check review box, signature, etc.) of review and verification of the relationship of each SAE to IP/study participation (causality) within 72 hours of SAE entry into the eCRF.<br>• After the study is completed at a given site, the electronic data collection tool will be taken off-line to prevent the entry of new data or changes to existing data.<br>• If a site receives a report of a new SAE from a study participant or receives updated data on a previously reported SAE after the electronic data collection tool has been taken off-line, then the site can report this information on a paper SAE form (see next section) or to the SAE coordinator by telephone.<br>• Contacts for SAE reporting can be found at the beginning of this protocol on the Sponsor/Medical Monitor Contact Information page. |

| SAE Reporting to GSK via Paper CRF |
| --- |
| • Facsimile transmission of the SAE paper CRF is the preferred method to transmit this information to the **SAE coordinator**.<br>• In rare circumstances and in the absence of facsimile equipment, notification by telephone is acceptable with a copy of the SAE data collection tool sent by overnight mail or courier service.<br>• Initial notification via telephone does not replace the need for the investigator to complete and sign the SAE CRF pages within the designated reporting time frames.<br>• Contacts for SAE reporting can be found at the beginning of this protocol on the Sponsor/Medical Monitor Contact Information page.. |

# 12.5. Appendix 5: Contraceptive Guidance and Collection of Pregnancy Information

## Definitions

### Woman of Childbearing Potential (WOCBP)

A woman is considered fertile following menarche and until becoming post-menopausal unless permanently sterile (see below)

**Women in the following categories are not considered WOCBP**

1. Premenarchal
2. Premenopausal female with ONE of the following:

- Documented hysterectomy
- Documented bilateral salpingectomy
- Documented bilateral oophorectomy

Note: Documentation can come from the site personnel's: review of participant's medical records, medical examination, or medical history interview.

3. Postmenopausal female

- A postmenopausal state is defined as no menses for 12 months without an alternative medical cause. A high follicle stimulating hormone (FSH) level in the postmenopausal range may be used to confirm a postmenopausal state in women not using hormonal contraception or HRT. However, in the absence of 12 months of amenorrhea, a single FSH measurement is insufficient.
- Females on HRT and whose menopausal status is in doubt will be required to use one of the non-hormonal highly effective contraception methods if they wish to continue their HRT during the study. Otherwise, they must discontinue HRT to allow confirmation of postmenopausal status before study enrolment.

## Contraception Guidance

### Female participants

Female participants of childbearing potential are eligible to participate if they agree to use a highly effective method of contraception consistently and correctly as described in Table 7.

**Table 7 Highly Effective Contraceptive Methods**

| |
|---|
| **Highly Effective Contraceptive Methods That Are User Dependent** [a]<br>*Failure rate of <1% per year when used consistently and correctly.* |
| Combined (estrogen- and progestogen-containing ) hormonal contraception associated with inhibition of ovulation[b]<br>• oral<br>• intravaginal<br>• transdermal |
| Progestogen-only hormonal contraception associated with inhibition of ovulation[b]<br>• injectable |
| **Highly Effective Methods That Are User Independent** |
| • Implantable progestogen-only hormonal contraception associated with inhibition of ovulation[b]<br>• Intrauterine device<br>• Intrauterine hormone-releasing system<br>• bilateral tubal occlusion |
| Vasectomized partner<br>*(A vasectomized partner is a highly effective contraception method provided that the partner is the sole male sexual partner of the WOCBP and the absence of sperm has been confirmed. If not, an additional highly effective method of contraception should be used.)* |
| Sexual abstinence<br>(*Sexual abstinence is considered a highly effective method only if defined as refraining from heterosexual intercourse during the entire period of risk associated with the study drug. The reliability of sexual abstinence needs to be evaluated in relation to the duration of the study and the preferred and usual lifestyle of the participant.)* |

NOTES:

a. Typical use failure rates may differ from those when used consistently and correctly. Use should be consistent with local regulations regarding the use of contraceptive methods for participants in clinical studies.

b. Hormonal contraception may be susceptible to interaction with the study drug, which may reduce the efficacy of the contraceptive method. In this case two highly effective methods of contraception should be utilized during the treatment period and for the follow-up period until the last visit at Week 24 (or 16 weeks after the last dose, which is equivalent to 5.5 half-lives).

### Pregnancy Testing

- WOCBP should only be included after a confirmed menstrual period and a negative highly sensitive serum pregnancy test
- Additional pregnancy testing during the treatment period as specified in the SoA (Section 2).

- Pregnancy testing will be performed whenever a menstrual cycle is missed or when pregnancy is otherwise suspected
- Pregnancy testing will be performed and assayed in a certified laboratory OR and assayed in the central laboratory OR using the test kit provided by the central laboratory / provided by the sponsor /approved by the sponsor and in accordance with instructions provided in its package insert

## Collection of Pregnancy Information

### Female Participants who become pregnant

- Investigator will collect pregnancy information on any female participant, who becomes pregnant while participating in this study.
- Information will be recorded on the appropriate form and submitted to GSK within 24 hours of learning of a participant's pregnancy.
- Participant will be followed to determine the outcome of the pregnancy. The investigator will collect follow up information on participant and neonate, which will be forwarded to GSK Generally, follow-up will not be required for longer than 6 to 8 weeks beyond the estimated delivery date.
- Any termination of pregnancy will be reported, regardless of foetal status (presence or absence of anomalies) or indication for procedure.
- While pregnancy itself is not considered to be an AE or SAE, any pregnancy complication or elective termination of a pregnancy will be reported as an AE or SAE.
- A spontaneous abortion is always considered to be an SAE and will be reported as such.
- Any SAE occurring as a result of a post-study pregnancy which is considered reasonably related to the study treatment by the investigator, will be reported to GSK as described in Appendix 4. While the investigator is not obligated to actively seek this information in former study participants, he or she may learn of an SAE through spontaneous reporting.

Any female participant who becomes pregnant while participating will discontinue study treatment.

## 12.6. Appendix 6: Genetics

### USE/ANALYSIS OF DNA

- Genetic variation may impact a participant's response to therapy, susceptibility, severity and progression of disease. Variable response to therapy may be due to genetic determinants that impact drug absorption, distribution, metabolism, and excretion; mechanism of action of the drug; disease etiology; and/or molecular subtype of the disease being treated. Therefore, where local regulations and IRB/IEC allow, a blood sample will be collected for DNA analysis
- DNA samples will be used for research related to GSK3772847 or asthma and related diseases. They may also be used to develop tests/assays including diagnostic tests) related to GSK3772847 or other treatments which may regulate eosinophils, impact the epithelium or other study treatments including, but not limited to, steroids, long-acting beta-agonists, and other drugs used in the treatment of asthma, or for asthma and related diseases. Genetic research may consist of the analysis of one or more candidate genes or the analysis of genetic markers throughout the genome or analysis of the entire genome (as appropriate)
- DNA samples may be analysed for genetic effects on response. This may include, but not be limited to, an investigation as to whether polymorphisms from IL33 and IL1RL1 gene regions associate with IL33 or soluble ST2 expression levels or associate with efficacy or safety responses. Additional analyses may be conducted if it is hypothesized that this may help further understand the clinical data.
- The samples may be analysed as part of a multi-study assessment of genetic factors involved in the response to GSK3772847 or study treatments of this class. The results of genetic analyses may be reported in the clinical study report or in a separate study summary.
- The sponsor will store the DNA samples in a secure storage space with adequate measures to protect confidentiality.
- The samples will be retained while research on GSK3772847 (or study treatments of this class) or asthma continues but no longer than 15 years after the last subject last visit or other period as per local requirements.

## 12.7. Appendix 7: Liver Safety: Required Actions and Follow-up Assessments

**Phase II liver chemistry stopping and increased monitoring criteria** have been designed to assure participant safety and evaluate liver event aetiology

**Phase II liver chemistry stopping criteria and required follow up assessments**

| **Liver Chemistry Stopping Criteria** | |
|---|---|
| **ALT-absolute** | ALT ≥5xULN |
| **ALT Increase** | ALT ≥3xULN persists for ≥4 weeks |
| **Bilirubin[1, 2]** | ALT ≥3xULN **and** bilirubin ≥2xULN (>35% direct bilirubin) |
| **INR[2]** | ALT ≥3xULN **and** INR>1.5, if INR measured |
| **Cannot Monitor** | ALT ≥3xULN and cannot be monitored weekly for 4 weeks |
| **Symptomatic[3]** | ALT ≥3xULN associated with symptoms (new or worsening) believed to be related to liver injury or hypersensitivity |

| **Required Actions and Follow up Assessments** | |
|---|---|
| **Actions** | **Follow Up Assessments** |
| • **Immediately** discontinue study treatment<br>• Report the event to GSK **within 24 hours**<br>• Complete the liver event CRF and complete an SAE data collection tool if the event also meets the criteria for an SAE[2]<br>• Perform liver chemistry event follow up assessments<br>• Monitor the participant until liver chemistries resolve, stabilise, or return to within baseline (see **MONITORING** below)<br>• **Do not restart/rechallenge** participant with study treatment unless allowed per protocol and GSK Medical Governance approval **is** granted (see below)<br>If restart/rechallenge **not allowed per protocol or not granted**, permanently discontinue study treatment and continue participant in the study for any protocol specified follow up assessments | • Viral hepatitis serology[4]<br>• Obtain INR and recheck with each liver chemistry assessment until the transaminases values show downward trend<br>• Obtain blood sample for PK analysis winthin 1 week after the liver event[5]<br>• Serum CPK and lactate dehydrogenase.<br>• Fractionate bilirubin, if total bilirubin≥2xULN<br>• Obtain complete blood count with differential to assess eosinophilia<br>• Record the appearance or worsening of clinical symptoms of liver injury, or hypersensitivity, on the AE report form<br>• Record use of concomitant medications on the concomitant medications report |

| | |
|---|---|
| **MONITORING:**<br><br>**<u>For bilirubin or INR criteria:</u>**<br><br>• Repeat liver chemistries (include ALT, AST, alkaline phosphatase, bilirubin) and perform liver event follow up assessments within **24 hrs**<br><br>• Monitor participants twice weekly until liver chemistries resolve, stabilise or return to within baseline<br><br>• A specialist or hepatology consultation is recommended<br><br>**<u>For All other criteria:</u>**<br><br>• Repeat liver chemistries (include ALT, AST, alkaline phosphatase, bilirubin) and perform liver event follow up assessments within **24-72 hrs**<br><br>• Monitor participants weekly until liver chemistries resolve, stabilise or return to within baseline | form including acetaminophen, herbal remedies, other over the counter medications.<br><br>• Record alcohol use on the liver event alcohol intake case report form (CRF) page<br><br>**<u>For bilirubin or INR criteria:</u>**<br><br>• Anti-nuclear antibody, anti-smooth muscle antibody, Type 1 anti-liver kidney microsomal antibodies, and quantitative total Immunoglobulin G or gamma globulins.<br><br>• Serum acetaminophen adduct high performance liquid chromatography (HPLC) assay (quantifies potential acetaminophen contribution to liver injury in participants with definite or likely acetaminophen use in the preceding week [James, 2009]).<br><br>• Liver imaging (ultrasound, magnetic resonance, or computerised tomography) and /or liver biopsy to evaluate liver disease; complete Liver Imaging and/or Liver Biopsy CRF pages. |

1. Serum bilirubin fractionation should be performed if testing is available. If serum bilirubin fractionation is not immediately available, discontinue study treatment for that subject if ALT ≥3xULN **and** bilirubin ≥2xULN. Additionally, if serum bilirubin fractionation testing is unavailable, **record presence of detectable urinary bilirubin on dipstick**, indicating direct bilirubin elevations and suggesting liver injury.
2. All events of ALT ≥3xULN **and** bilirubin ≥2xULN (>35% direct bilirubin) or ALT ≥3xULN **and** INR>1.5, if INR measured which may indicate severe liver injury (possible 'Hy's Law'), **must be reported as an SAE (excluding studies of hepatic impairment or cirrhosis)**; INR measurement is not required and the threshold value stated will not apply to participants receiving anticoagulants
3. New or worsening symptoms believed to be related to liver injury (such as fatigue, nausea, vomiting, right upper quadrant pain or tenderness, or jaundice) or believed to be related to hypersensitivity (such as fever, rash or eosinophilia)
4. Includes: Hepatitis A IgM antibody; Hepatitis B surface antigen (HbsAg) and Hepatitis B Core Antibody (IgM); Hepatitis C RNA; Cytomegalovirus IgM antibody; Epstein-Barr viral capsid antigen IgM antibody (or if unavailable, obtain heterophile antibody or monospot testing); Hepatitis E IgM antibody
5. PK sample may not be required for participants known to be receiving placebo or non-GSK comparator treatments.) Record the date/time of the PK blood sample draw and the date/time of the last dose of study treatment prior to PK blood sample draw on the CRF. If the date or time of the last dose is unclear, provide the participant's best approximation. If the date/time of the last dose cannot be approximated OR a PK sample cannot be collected in the time period indicated above, do not obtain a PK sample. Instructions for sample handling and shipping are in the SRM.

**Phase II liver chemistry increased monitoring criteria with continued therapy**

| Liver Chemistry Increased Monitoring Criteria – Liver Monitoring Event | |
|---|---|
| **Criteria** | **Actions** |
| ALT ≥3xULN and <5xULN **and** bilirubin <2xULN**, without** symptoms believed to be related to liver injury or hypersensitivity, **and** who can be monitored weekly for 4 weeks | • Notify the GSK medical monitor **within 24 hours** of learning of the abnormality to discuss participant safety.<br>• Participant can continue study treatment<br>• Participant must return weekly for repeat liver chemistries (ALT, AST, alkaline phosphatase, bilirubin) until they resolve, stabilise or return to within baseline<br>• If at any time participant meets the liver chemistry stopping criteria, proceed as described above<br>• If, after 4 weeks of monitoring, ALT <3xULN and bilirubin <2xULN, monitor participants twice monthly until liver chemistries normalize or return to within baseline. |

**References**

James LP, Letzig L, Simpson PM, Capparelli E, Roberts DW, Hinson JA, Davern TJ, Lee WM. Pharmacokinetics of Acetaminophen-Adduct in Adults with Acetaminophen Overdose and Acute Liver Failure. Drug Metab Dispos 2009; 37:1779-1784.

## 12.8. Appendix 8 : Protocol Amendment History

The Protocol Amendment Summary of Changes Table for the current amendment is located directly before the Table of Contents (TOC).

**Amendment 1** 05-FEB-2018

**Overall Rationale for the Amendment:** To address clarifications regarding the eligibility criteria, the study population, the schedule of activities and the clinical assessments. Also, a few typographical errors were corrected.

| Section # and Name | Description of Change | Brief Rationale |
|---|---|---|
| Section 6.2 Table 2;<br>Section 7.7.2 | Clarified that oral corticosteroids are not permitted as standard of care. | Participants with severe asthma for whom it is felt oral corticosteroids are warranted are not eligible for this study. |
| Section 6.2 Table 2;<br>Section 7.7.1 | Updated Section 7.7.1 to support the use of anti-fungal medication. Removed anti-fungal medication from the list of prohibited medications in Table 2. | The study is being stratified on the use of anti-fungal medication and therefore treatment with anti-fungal medication is permitted during the treatment period if used in a consistent dosage and frequency from screening and throughout the treatment period. |
| Section 3.1 Study Rationale | Clarified that the study population may include participants who have asthma with allergic bronchopulmonary aspergillosis (ABPA). | The target population for this study is moderate to severe asthmatics who have allergic fungal airway disease (AFAD). AFAD is determined by positive IgE sensitisation to thermotolerant filamentous fungi particularly Aspergillus fumigatus. Therefore, this definition would include participants who have asthma and ABPA as ABPA is loosely defined by the following features: eosinophilia, fleeting lung shadows, high total IgE, and raised A fumigatus–specific IgE. |
| Section 1 Synopsis;<br>Section 4 Objectives and Endpoints;<br>Section 9.4.2 Vital Signs;<br>Section 10.3.2 Safety Analyses | Removed the body temperature in the objectives and safety analyses and updated Section 9 to reflect that body temperature will be collected but only available in source documents. | Body temperature will be monitored at the site only and not included in any analyses. |
| Section 2 Schedule of Activities | Updated the note as to the timing for obtaining informed consent. | Informed consent may be obtained prior to Screening. |

| Section # and Name | Description of Change | Brief Rationale |
|---|---|---|
| Section 2 Schedule of Activities | Updated the definition of the medical history notes. | Medical history should include asthma disease duration and exacerbation history. |
| Section 2 Schedule of Activities | Clarified the notes for sputum sample collection. | If a participant does not produce a viable baseline sputum sample at visit 2 or at visit 3, the participant will not need to undergo a sputum induction at the end of the treatment period. |
| Section 2 Schedule of Activities | Changed the PK sample timepoint 'post end of infusion' to 'post dose'. | Clarify the definition of the post dose sample such that it is consistent with Section 2 notes. |
| Section 2 Schedule of Activities | Clarified the Vital signs notes as to order of performance. | Vital signs should be obtained prior to the 12-lead ECGs. |
| Section 2 Schedule of Activities;<br>Section 9.2.1 Time Period and Frequency for Collecting AE and SAE Information | Adjusted the timing for collection of AEs/SAEs from randomisation onward and for collection of SAEs at V1 and V2. | At Visits 1 and 2 SAE information will only be collected if considered as related to study participation. AEs and SAEs will be collected from randomisation until the end of the study. |
| Section 3 Introduction;<br>Section 11 References | Added newly published internal reference and corrected the dates for the referenced Ph2 study. | Added newly available clinical study report reference and accurate dates of the Ph2 study with the same IP. |
| Section 3.3.1 Risk Assessment;<br>Section 10.3.6 Interim Analyses;<br>Appendix 1 Abbreviations and Trademarks<br>Appendix 3 Study Governance Considerations | Changed independent safety review committee to internal safety review committee (iSRC).<br>Added a link to Appendix 3 in reference to the iSRC. | The safety review committee is independent of the study team but does have members who are employees of the sponsor. The title of the committee was changed to reflect its affiliation to the sponsor. The description of the iSRC is included in Appendix 3. |
| Section 6.1 Inclusion Criteria;<br>Appendix 5 Contraceptive Guidance and Collection of | Updated Inclusion criterion 9aii and Table 7 Note b with regards to the contraceptive | A WOCBP will be eligible if they agree to follow the contraceptive guidance in |

| Section # and Name | Description of Change | Brief Rationale |
|---|---|---|
| Pregnancy Information | requirements for female participants. | Appendix 5 during the treatment period and for the duration of the follow-up period until the last visit at week 24 (or 16 weeks after the last dose, which is equivalent to 5.5 half-lives). |
| Section 6.2 Exclusion Criteria | Provided a definition for former smokers. | Former smokers are defined as smokers with greater than 6 months abstention. |
| Section 6.3 Run-in Exclusion Criteria;<br>Section 6.5 Randomisation Exclusion Criteria | Clarified and corrected the run-in and randomisation criteria sections. | Removed criteria in each section pertaining to the previous visits or sections and corrected typographical errors and visit label within each section. |
| Section 6.7 Screening/Run-in/Randomisation Failures | Updated the circumstances allowed for rescreening and the screening number for a rescreened participant. | If a participant has an asthma exacerbation at the time of run-in or randomisation, they may be rescreened at the Investigator's discretion in consulation with the sponsor after the exacerbation has resolved. |
| Section 8.1 Discontinuation of Study Treatment | Clarified the stopping criteria for study treatment to align with Section 7.4 Blinding. | In the event of unblinding by the investigator or treating physician the Medical monitor/GSK team should be contacted to determine whether subject withdrawal is required. |
| Section 1 Synopsis | Deleted a space between *fumigatus* and the period. | Typographical error |
| Section 2 Schedule of Activities | Updated the sST2 boxes to match the assessments. | Formatting error |
| Section 3.2 Background | Corrected spelling of the term 'thermotolerant' | Typographical error |
| Section 5.2 Number of Participants | Corrected the spelling of randomised. | Typographical error |

| Section # and Name | Description of Change | Brief Rationale |
|---|---|---|
| Section 6.1 Inclusion criteria | Added a parentheses after ‘guidelines’ in inclusion criterion 2 | Typographical error |
| Section 6.2 Exclusion criteria | Replaced word ‘on’ with ‘of’ in the exclusion criteria 9. | Typographical error |
| Section 6.3 Run-in Exclusion Criteria | Corrected the direct bilirubin levels to >35%. | Typographical error |
| Section 10.2 Populations for Analyses | Changed the word subject to participant and corrected randomised. | Typographical errors |
| Appendix 3 Study Governance Considerations, Dissemination of Clinical Study Data | Corrected the spelling of randomisation | Typographical error |

## 12.9 Appendix 9: FRENCH-SPECIFIC REQUIREMENTS

This appendix includes all applicable requirements of French Public Health Code / specific local GSK requirements and identifies, item per item, the mandatory modifications or additional information to the study protocol.

**1. Concerning the « SELECTION OF STUDY POPULATION AND WITHDRAWAL CRITERIA»**

- Subjects will be compensated for the inconvenience of participating in the study. The amount of compensation is stated in the Informed Consent Form. Subjects not completing the study for whatever reason could be compensated generally on a pro rata basis.

- According to French Public Health Code law (L.1121-16 and R.1121-16), the following people must be registered in National File ("Fichier National"):
  - Healthy volunteer ;
  - Subjects if the aim of the study is not linked to their disease;
  - Subjects on request of the Ethics Committee regarding study risks and constraints.

  The following details will be described:
  - Reference of the study
  - Surname and first name
  - Date and place of birth
  - Gender
  - Dates of beginning and termination of the study
  - Exclusion period during which the subject cannot participate to another study (French Public Health Code law L.1121-12)
  - The total amount of compensation

  The subjects' registration in National File ("Fichier National") should be documented in the source document - subject notes and monitored by the CRA.

- The following vulnerable subject populations will be excluded: minors, protected subjects, adult subjects not in condition to express their consent, subjects deprived of liberty, subjects receiving psychiatric cares, subjects hospitalized in a Health and Social Establishment for other purpose than the participation to the study.

- A subject will be eligible for inclusion in this study if he /she is either affiliated to or beneficiary of a social security category (French Public Health Code law L.1121-8-1). (exception for a participant to a non-interventional study if authorised by the Ethics Committee).

  It is the investigator's responsibility to ensure and to document (in the source document - subject notes) that the subject:
  - is either affiliated to or beneficiary of a social security category ;
  - has got an authorisation by the Ethics Committee.

**2. Concerning the "STATISTICAL CONSIDERATIONS AND DATA ANALYSES" and specially in the "SAMPLE SIZE ASSUMPTIONS"**

The expected number of subjects to be recruited in France is declared to the French regulatory authority.

## 3. Concerning the “STUDY GOVERNANCE CONSIDERATIONS”

- **In section “Regulatory and Ethical Considerations, including the Informed Consent Process”**

⇒ Concerning **the process for informing the subject** and/or his/her legally authorized representative

French Patient Informed Consent Form is a document which summarizes the main features of the study and allows collection of the subject and/or his/her legally authorized representative written consent in triplicate (quadriplicate for minor subject). It also contains a reference to the authorisation of ANSM and the approval from the French Ethics Committee and the maintenance of confidentiality of the returned consent form by GlaxoSmithKline France.

⇛ **Concerning the management of the Patient Informed Consent Forms**

The first copy of the Patient Informed Consent Form is kept by the investigator. The second copy is kept by the Medical Direction of GlaxoSmithKline France and the last copy(ies) is(are) given to the subject or their legally authorized representative(s).

The second copy of all the consent forms will be collected by the Clinical Research Assistant (CRA) under the’s the Investigator’s control, and placed in a sealed envelope bearing only:
- the study number,
- the identification of the Centre: name of the principal investigator and centre number,
- the number of informed consents,
- the date,
- and the principal investigator’s signature.

Then, the CRA hands the sealed envelope over to the Medical Direction, for confidential recording, under the responsibility of the Medical Director.

- **NOTIFICATION TO THE HOSPITAL DIRECTOR**

In accordance with Article L1123-13 of the French Public Health Code, the Hospital Director is informed of the commitment to the trial in her/his establishment. The Hospital Director is supplied with the protocol and any information needed for the financial disposition, the name of the investigator(s), the number of sites involved in his establishment and the estimated time schedule of the trial (R.1123-69).

- **INFORMATION TO THE HOSPITAL PHARMACIST**

In accordance with Article R.1123-70 of the French Public Health Code, the Hospital Pharmacist is informed of the commitment to the trial in her/his establishment. The Pharmacist is supplied with a copy of the protocol (which allows her/him to dispense the drug(s) of the trial according to the trial methodology), all information concerning the product(s) of the trial (e.g. included in the CIB), the name of the investigator(s), the number of sites involved in her/his establishment and the estimated time schedule of the trial.

## 4. Concerning the "DATA MANAGEMENT"

- Within the framework of this clinical trial, data regarding the identity of the investigators and/or co-investigators and/or the pharmacists if applicable, involved in this clinical trial, and data regarding the subjects recruited in this clinical trial (subject number, treatment number, subjects status with respect to the clinical trial, dates of visit, medical data) will be collected and computerized in GlaxoSmithKline data bases by GlaxoSmithKline Laboratory or on its behalf, for reasons of follow up, clinical trial management and using the results of said clinical trial. According to the Data Protection French Law n° 78-17 of 6$^{th}$ January 1978, each of these people aforesaid has a right of access, correction and opposition on their own data through GlaxoSmithKline Laboratory (Clinical Operations Department).

- **DEMOGRAPHIC DATA**

In accordance with the Data Protection French Law n° 78-17 of 6$^{th}$ January 1978 – article 8, the ethnic origin can only be collected if the collection of this data is strictly necessary and relevant for the purpose of the study.

- **TESTING OF BIOLOGICAL SAMPLES**

In accordance with the French Public Health Code law – article L1211-2, a biological sample without identified purpose at the time of the sample and subject's preliminary information is not authorized.

## 5. SAE

In case of paper notification, the SAE Reports have to be transmitted to the GlaxoSmithKline France Drug Safety Department, which name, address and phone number are:

**Département de Pharmacovigilance**
**Laboratoire GlaxoSmithKline**
**23, rue François Jacob**
**92 500 Rueil-Malmaison**
**Tel :** PPD
**Fax :** PPD
PPD

## 6. Monitoring visits

The Health Institution and the Investigator agree to receive on a regular basis a Clinical Research Assistant (CRA) of GLAXOSMITHKLINE or of a service provider designated by GLAXOSMITHKLINE. The Health Institution and the Investigator agree to be available for any phone call and to systematically answer to all correspondence regarding the Study from GLAXOSMITHKLINE or from a service provider designated by GLAXOSMITHKLINE. In addition, the Health Institution and the Investigator agree that the CRA or the service provider designated by GLAXOSMITHKLINE have direct access to all the data concerning the Study (test results, medical record, etc …). This consultation of the information by GLAXOSMITHKLINE is required to validate the data registered in the electronic Case Report Form (eCRF), in particular by comparing them directly to the source data. In accordance with the legal and regulatory requirements, the strictest confidentiality will be respected.

## 7. Data entry into the eCRF

The Health Institution and the Investigator agree to meet deadlines, terms and conditions of the Study's electronic Case Report Form (eCRF) use here below:

The Health Institution and the Investigator undertake:

1. That the Investigator and the staff of the investigator center make themselves available to attend the training concerning the computer system dedicated to the electronic Case Report Form (eCRF) of the Study provided by GLAXOSMITHKLINE or by a company designated by GLAXOSMITHKLINE.
2. That the Investigator and the staff of the investigator center use the IT Equipment loaned and/or the access codes only for the purpose of which they are intended and for which they have been entrusted to them, namely for the Study achievement, to the exclusion of any other use.
3. That the Investigator and the staff of the investigator center use the IT Equipment loaned according to the specifications and manufacturer's recommendations which will have been provided by GLAXOSMITHKLINE.
4. To keep the IT Equipment and/or access codes in a safe and secure place and to authorize only the use of this IT Equipment by investigator center staff designated by the principal investigator to enter the data of the Study.
5. To be responsible for the installation and payment of the required Internet connections needed for the use of the IT Equipment, Computer systems and/or access codes.
6. To return at the end of the Study the IT Equipment and/or access codes to GLAXOSMITHKLINE or to any company designated by GLAXOSMITHKLINE and any training material and documentation. The IT Equipment cannot under any circumstances be kept by the Health Institution or the Investigator for any reason whatsoever.

## 8. CTR publication

It is expressly specified that GLAXOSMITHKLINE and/or the Sponsor can make available to the public the results of the Study by the posting of the said results on a website of the GLAXOSMITHKLINE GROUP named Clinical Trial Registred (CTR) including the registration of all the clinical trials conducted by the GLAXOSMITHKLINE Group and this before or after the publication of such results by any other process.

## 9. Data Protection French Law of 6th January 1978 (CNIL)

In accordance with the Data Protection French Law of 6 January 1978, computer files used by GLAXOSMITHKLINE to monitor and to follow the implementation and the progress of the Study are declared with CNIL by GLAXOSMITHKLINE. The Investigator has regarding the processing data related to her/him a right of access, of rectification and of opposition with GLAXOSMITHKLINE in accordance with the legal provisions. This information can be transferred or be accessed to other entities of GLAXOSMITHKLINE Group, what the Investigator agrees by the signature of the present Protocol.

## 10. Investigational Product Accountability, Reconciliation, and Destruction

In specific situations (if the site has 1- an approval from the French Regulatory Agency (ANSM) and 2- a GSK written endorsement letter) where institutional practices dictate that the site disposes of and/or destroys IP prior to allowing the “monitor’ to verify and document IP accountability, the following applies:
*“During the conduct of the Study, Investigational Product (IP) will be destroyed by the Institution prior to a GlaxoSmithKline “**monitor**” conducting final investigational product accountability. Institution agrees that such destruction will comply with Institution’s investigational product accountability procedures and will provide GlaxoSmithKline with investigational product accountability logs and supporting documentation to verify adherence to ‘Bonnes Pratiques Cliniques’ (decision dated on the 24th of November 2006).*
