# Supplementary material for: Efficacy and safety of GSK3772847 in participants with moderate-to-severe asthma with allergic fungal airway disease: A phase IIa randomized, multicenter, double-blind, sponsor-open, comparative trial
Source: PLoS One. 2023 Feb 3;18(2):e0281205. doi: 10.1371/journal.pone.0281205 (PMC9897512; doi:10.1371/journal.pone.0281205)
Supplement: S1 Table — (DOCX) [file pone.0281205.s003.docx]

**S3 Table. Raw data for Fig 2: Ratio to baseline in free soluble ST2 levels*.**

|  | Placebo (n=9) | | | | GSK233705 (n=8) | | | |
| --- | --- | --- | --- | --- | --- | --- | --- | --- |
|  | Week 4 (Pre-dose) | Week 8  (pre-dose) | Week 12 | Week 24 | Week 4 (Pre-dose) | Week 8 (pre-dose) | Week 12 | Week 24 |
| n | 8 | 8 | 8 | 9 | 8 | 8 | 7 | 8 |
| Ratio to baseline, Geometric mean  (95% CI) | 0.884 (0.735, 1.063) | 0.951 (0.740, 1.224) | 0.800 (0.610, 1.049) | 0.844 (0.664, 1.072) | 0.082 (0.064, 0.104) | 0.075 (0.048, 0.118) | 0.053 (0.030, 0.094) | 0.500 (0.292, 0.855) |

*This table includes on- and off-treatment data (on-treatment defined window is 28 days).
CI, confidence interval; ST2, suppressor of tumorigenicity 2.
